# Individualised prediction of drug resistance and seizure recurrence after medication withdrawal in people with juvenile myoclonic epilepsy: A systematic review and individual participant data meta-analysis

**DOI:** 10.1016/j.eclinm.2022.101732

**Published:** 2022-11-11

**Authors:** Remi Stevelink, Dania Al-Toma, Floor E. Jansen, Herm J. Lamberink, Ali A. Asadi-Pooya, Mohsen Farazdaghi, Gonçalo Cação, Sita Jayalakshmi, Anuja Patil, Çiğdem Özkara, Şenay Aydın, Joanna Gesche, Christoph P. Beier, Linda J. Stephen, Martin J. Brodie, Gopeekrishnan Unnithan, Ashalatha Radhakrishnan, Julia Höfler, Eugen Trinka, Roland Krause, EpiPGX Consortium, Emanuele Cerulli Irelli, Carlo Di Bonaventura, Jerzy P. Szaflarski, Laura E. Hernández-Vanegas, Monica L. Moya-Alfaro, Yingying Zhang, Dong Zhou, Nicola Pietrafusa, Nicola Specchio, Giorgi Japaridze, Sándor Beniczky, Mubeen Janmohamed, Patrick Kwan, Marte Syvertsen, Kaja K. Selmer, Bernd J. Vorderwülbecke, Martin Holtkamp, Lakshminarayanapuram G. Viswanathan, Sanjib Sinha, Betül Baykan, Ebru Altindag, Felix von Podewils, Juliane Schulz, Udaya Seneviratne, Alejandro Viloria-Alebesque, Ioannis Karakis, Wendyl J. D'Souza, Josemir W. Sander, Bobby P.C. Koeleman, Willem M. Otte, Kees P.J. Braun

**Affiliations:** a Department of Child Neurology, UMC Utrecht Brain Center, University Medical Center Utrecht, European Reference Network EpiCARE, Heidelberglaan 100, Utrecht, 3584 CX, Netherlands; b Department of Genetics, Center for Molecular Medicine, University Medical Center Utrecht, European Reference Network EpiCARE, Heidelberglaan 100, Utrecht, 3584 CX, Netherlands; c Epilepsy Research Center, Shiraz University of Medical Sciences, Zand, Shiraz, Iran; d Department of Neurology, Thomas Jefferson University, 909 Walnut Street, Philadelphia, PA, 19107, USA; e Department of Neurology, Unidade Local de Saude do Alto Minho, Estrada de Santa Luzia, Viana do Castelo, 4904-858, Portugal; f Department of Neurology, Krishna Institute of Medical Sciences, Minister Road, Secunderabad, 500003, India; g Department of Neurology, Cerrahpasa Faculty of Medicine, Istanbul University-Cerrahpaşa, Kocamustafapaşa caddesi, Istanbul, 34098, Turkey; h Department of Neurology, Yedikule Chest Diseases and Chest Surgery Training and Research Hospital, University of Health Sciences, Belgrat Kapı yolu, Istanbul, 34020, Turkey; i Department of Neurology, Odense University Hospital, J.B. Winsløws Vej 4, Odense, 5000, Denmark; j Department of Clinical Research, University of Southern Denmark, J.B. Winsløws Vej 4, Odense, 5000, Denmark; k Epilepsy Unit, University of Glasgow, University Avenue, Glasgow, G12 8QQ, UK; l Department of Neurology, R. Madhavan Nayar Center for Comprehensive Epilepsy Care, Sree Chitra Tirunal Institute for Medical Sciences and Technology, Chalakkuzhi, Medical College Road, Trivandrum, 695011, India; m Department of Neurology and Neuroscience Institute, Christian Doppler Medical Centre, Paracelsus Medical University and Centre for Cognitive Neuroscience, European Reference Network EpiCARE, Ignaz-Harrer Straße 79, Salzburg, 5020, Austria; n Karl Landsteiner Institute for Neurorehabilitation and Space Neurology, Hellbrunner Straße 34, Salzburg, 3100, Austria; o Department of Public Health, University for Health Sciences, Medical Informatics and Technology, Eduard-Wallnöfer-Zentrum 1, Hall in Tirol, 6060, Austria; p Bioinformatics Core Facility, Luxembourg Centre for Systems Biomedicine, University of Luxembourg, 6 Ave du Swing, Belvaux, 4367, Luxembourg; q Department of Human Neurosciences, Epilepsy Unit, Sapienza, University of Rome, Viale dell'Università 30, Rome, 00185, Italy; r Departments of Neurology, Neurosurgery, and Neurobiology, UAB Epilepsy Center, University of Alabama at Birmingham Heersink School of Medicine, 1670 University Blvd, Birmingham, AL, 35294, USA; s Department of Clinical Research, Epilepsy Clinic, National Institute of Neurology and Neurosurgery, Insurgentes Sur 3877, Mexico, 14269, Mexico; t Department of Neurology, West China Hospital of Sichuan University, 37 Guoxue Road, Chengdu, 610000, China; u Department of Neuroscience, Division of Neurology, Bambino Gesù Children's Hospital, IRCCS, Piazza Sant'Onofrio, 4, Rome, 00165, Italy; v Department of Clinical Neurophysiology, Institute of Neurology and Neuropsychology, 83/11 Vazha-Pshavela Ave., Tbilisi, 186, Georgia; w Department of Clinical Neurophysiology, Danish Epilepsy Centre, Filadelfia, Visby Allé 5, Dianalund, 4293, Denmark; x Department of Clinical Neurophysiology, Aarhus University Hospital and Aarhus University, Palle Juul-Jensens Blvd. 99, Aarhus, 8200, Denmark; y Department of Neurosciences, Central Clinical School, Monash University, 99 Commercial Road, Melbourne, Victoria, 3004, Australia; z Departments of Medicine and Neurology, Royal Melbourne Hospital, University of Melbourne, Grattan Street, Parkville, Victoria, Australia; aa Department of Neurology, Vestre Viken Hospital Trust, Dronninggata 28, Drammen, 3004, Norway; ab National Centre for Epilepsy & Department of Research and Innovation, Division of Clinical Neuroscience, Oslo University Hospital, G. F. Henriksens vei 29, Sandvika, 1337, Norway; ac Department of Neurology, Epilepsy-Center Berlin-Brandenburg, Charité - Universitätsmedizin Berlin, Charitéplatz 1, Berlin, 10117, Germany; ad Department of Neurology, National Institute of Mental Health and Neurosciences (NIMHANS), Hosur Road, Bangalore, 560029, India; ae Department of Neurology and Clinical Neurophysiology, Istanbul Faculty of Medicine, Istanbul University, Millet Cad, Istanbul, 34390, Turkey; af Department of Neurology, Istanbul Florence Nightingale Hospital, Abide-i Hürriyet Cad, Istanbul, 34381, Turkey; ag Department of Neurology, Epilepsy Center, University Medicine Greifswald, Sauerbruchstraße, Greifswald, 17489, Germany; ah Department of Medicine, St Vincent's Hospital Melbourne, The University of Melbourne, 55 Victoria Parade, Melbourne, Victoria, 3065, Australia; ai Department of Medicine, The School of Clinical Sciences at Monash Health, Monash University, Clayton Road, Melbourne, Victoria, 3168, Australia; aj Department of Neurology, Hospital General de la Defensa, Vía Ibérica 1, Zaragoza, 50009, Spain; ak Instituto de Investigación Sanitaria (IIS) Aragón, Avda. San Juan Bosco 13, Zaragoza, 50009, Spain; al Department of Neurology, Emory University School of Medicine, 49 Jesse Hill Jr. Drive SE, Office 335, Atlanta, GA, 30303, USA; am Stichting Epilepsie Instellingen Nederland (SEIN), Achterweg 7, Heemstede, Netherlands; an UCL Queen Square Institute of Neurology, Queen Square, London, WC1N 3BG, UK

**Keywords:** Juvenile myoclonic epilepsy, Prediction model, Refractory epilepsy, Drug resistance, Medication withdrawal, Remission, Multivariable prediction, JME, Seizure recurrence, Meta-analysis, Individual participant data

## Abstract

**Background:**

A third of people with juvenile myoclonic epilepsy (JME) are drug-resistant. Three-quarters have a seizure relapse when attempting to withdraw anti-seizure medication (ASM) after achieving seizure-freedom. It is currently impossible to predict who is likely to become drug-resistant and safely withdraw treatment. We aimed to identify predictors of drug resistance and seizure recurrence to allow for individualised prediction of treatment outcomes in people with JME.

**Methods:**

We performed an individual participant data (IPD) meta-analysis based on a systematic search in EMBASE and PubMed – last updated on March 11, 2021 – including prospective and retrospective observational studies reporting on treatment outcomes of people diagnosed with JME and available seizure outcome data after a minimum one-year follow-up. We invited authors to share standardised IPD to identify predictors of drug resistance using multivariable logistic regression. We excluded pseudo-resistant individuals. A subset who attempted to withdraw ASM was included in a multivariable proportional hazards analysis on seizure recurrence after ASM withdrawal. The study was registered at the Open Science Framework (OSF; https://osf.io/b9zjc/).

**Findings:**

Our search yielded 1641 articles; 53 were eligible, of which the authors of 24 studies agreed to collaborate by sharing IPD. Using data from 2518 people with JME, we found nine independent predictors of drug resistance: three seizure types, psychiatric comorbidities, catamenial epilepsy, epileptiform focality, ethnicity, history of CAE, family history of epilepsy, status epilepticus, and febrile seizures. Internal-external cross-validation of our multivariable model showed an area under the receiver operating characteristic curve of 0·70 (95%CI 0·68–0·72). Recurrence of seizures after ASM withdrawal (n = 368) was predicted by an earlier age at the start of withdrawal, shorter seizure-free interval and more currently used ASMs, resulting in an average internal-external cross-validation concordance-statistic of 0·70 (95%CI 0·68–0·73).

**Interpretation:**

We were able to predict and validate clinically relevant personalised treatment outcomes for people with JME. Individualised predictions are accessible as nomograms and web-based tools.

**Funding:**

MING fonds.

**Research in context**

**Evidence before this study**

A third of people with JME are drug-resistant, and three-quarters of individuals with JME who attempted to withdraw treatment after becoming seizure-free experienced a seizure recurrence. We last performed a systematic literature search in PubMed and Embase on 11 March 2021 by searching for ‘juvenile myoclonic epilepsy’ and ‘treatment outcome’ and various synonyms. We found no sufficiently powered multivariable analyses that assessed potential predictors of drug-resistant JME or predictors of seizure recurrence after ASM withdrawal.

**Added value of this study**

This individual participant data (IPD) meta-analysis (n = 2518) identified nine independent predictors of drug-resistant JME, seven previously reported, and two novel: ethnicity and family history of epilepsy. We found three predictors of seizure recurrence after treatment withdrawal. The strongest predictor for post-withdrawal seizure recurrence in JME – earlier age at the start of withdrawal – had an inverse direction of effect compared to other epilepsy types. We used these variables to create prediction models of drug-resistant JME and of seizure recurrence. Internal-external cross-validation showed robust predictive performance of our models.

**Implications of all the available evidence**

We created and validated prediction models, available as nomograms and web-based tools, to improve and personalise JME treatment. We expect that the models will aid in improving and personalising the treatment and counselling of people with JME.

## Introduction

Juvenile myoclonic epilepsy (JME) is the most common idiopathic and presumed genetic generalised epilepsy syndrome, affecting 5–10% of all people with epilepsy.^1^ Response to anti-seizure medication (ASM) is often assumed to be good,^2^^,^^3^ but we recently reported that a third of all people with JME are drug-resistant.^4^ People with JME are widely believed to require lifelong treatment.^5^^,^^6^ After a period of seizure freedom, however, around a quarter of those who withdraw treatment may remain seizure-free.^4^

Predicting who is likely to become drug-resistant and who could safely withdraw ASM treatment after a certain period of sustained seizure-freedom, has clinical benefits. Drug withdrawal improves quality of life by avoiding the adverse effects of potentially unnecessary treatment.^7^^,^^8^ We previously identified 43 reports providing treatment outcomes in cohorts of people with JME.^4^ We found six prognostic risk factors for drug resistance. Some of these risk factors are collinear, and it is unknown which have independent predictive value. Recent published multivariable prediction models of drug resistance had intrinsic limitations due to relatively small and heterogeneous cohorts, including different types of generalised epilepsy.^9^^,^^10^ There are currently no known risk factors for seizure relapse after ASM withdrawal in JME, other than those previously identified in the broader epilepsy population.^11^

We aimed to identify independent predictors of drug resistance and post-withdrawal relapse risk based on individual participant data (IPD) from previously published study cohorts. We developed and validated predictive tools to calculate these risks.

## Methods

### Search strategy and selection criteria

We performed a meta-analysis of individual participant data according to a pre-registered protocol (https://osf.io/b9zjc/). The methods and reporting are consistent with the PRISMA-IPD^12^ and TRIPOD statements.^13^ We systematically searched PubMed and EMBASE for articles published in English, Dutch, German, Spanish or French describing treatment outcomes in observational cohorts of people with JME, with no date restrictions. The literature search was last updated by RS on March 11, 2021, using the same search terms and study-level inclusion and exclusion criteria as in a previously published meta-analysis^4^: we included retrospective and prospective studies reporting on treatment outcomes of people with a diagnosis of JME. As individuals were diagnosed before the proposed consensus criteria,^5^ our diagnoses were primarily made according to descriptive criteria. JME is a distinctive syndrome characterised by juvenile-onset myoclonic seizures and generalised tonic-clonic seizures (GTCS), usually occurring after wakening and evoked by sleep deprivation, alcohol consumption, and especially a combination of irregular spike and wave discharges in the EEG.^2^ We excluded articles exclusively reporting on people with drug-resistant JME or in remission. We excluded drug trials as these could be biased towards people with drug-resistant JME. We used three individual-level inclusion criteria: (1) clinical diagnosis of JME, regardless of the diagnostic criteria used by the study,^5^ (2) at least one year of follow-up, and (3) available information regarding seizure outcome, with ASM use. People with pseudo-resistant epilepsy were excluded, i.e. seizures due to non-compliance, inadequate treatment or inadequate lifestyle regulation.^14^ People who had attempted to withdraw ASM after a period of seizure freedom were included in an analysis to assess predictors of seizure relapse after ASM withdrawal. We found one additional article by cross-referencing.

We invited the corresponding authors of all potentially eligible studies to collaborate by sharing IPD. If we received no reply, we sent two reminders 4–6 weeks apart, and when possible, we contacted additional authors of the same study. We searched ResearchGate, the International League Against Epilepsy (ILAE) website, other publications by the same authors and performed a manual internet search for alternative contact details.

Authors who agreed to collaborate were asked to provide treatment outcome data and potential predictors by filling in a standardised data entry sheet containing 41 variables (Supplementary Table S1). Alternatively, collaborators could send a datasheet in their format, after which the coordinating investigator standardised the data. Some collaborators could update their data with additional variables or individuals not included in their original publication. All datasets were manually reviewed, and potential discrepancies were resolved by discussion with the contributing author. We did not include aggregate study data without IPD.

As in our previous meta-analysis, we used the Newcastle–Ottawa quality assessment scale for cohort studies to assess the methodological quality of all the included studies.^15^ The scale ranges between 1 and 8, where higher scores represent a higher quality and less risk of bias.

Our study was a meta-analysis of de-identified individual data and did not require ethical approval or specific informed consent. Local research ethics committees or other entities overseeing personal data had approved the original studies. Where applicable under local regulations, data sharing agreements were signed before receiving individual data.

### Outcome and predictor variables

We used a combination of outcome measures to define drug resistance and seizure recurrence after ASM withdrawal. For the primary analysis, we used the definition of drug-resistant epilepsy formulated by the ILAE, taking each seizure type into account.^16^ This was defined as the failure of two or more adequate trials of well-tolerated and appropriately chosen drug schedules. We assessed whether people had not had seizures of any type in the last one, two or five years of follow-up as sensitivity analyses. Similarly, we specifically ascertained whether individuals were free of GTCS in the last one, two or five years of follow-up, as these are the most debilitating seizure type and are less likely to be underreported.

We assessed seizure recurrence in a subset of people who attempted to withdraw treatment after a period of seizure freedom. Seizure recurrence was evaluated at two and five years after initiation of ASM withdrawal. Our primary analysis comprised recurrence of any seizure after start of ASM withdrawal. We also specifically assessed GTCS recurrence.

We selected candidate predictors of drug resistance and seizure recurrence based on our previous meta-analysis on refractory JME,^4^ our previous publication on seizure recurrence in general cohorts of people with epilepsy,^11^ and potential predictors identified by included studies. We focused on readily available predictors in a routine clinical setting, excluding variables such as advanced EEG processing data or functional MRI biomarkers. Supplementary Table S1 provides an overview of all outcome measures, predictors, and definitions.

### Data analysis

The supplementary methods provide a detailed overview of all analyses and statistical methods. In brief, the proportion of drug-resistant JME was assessed with random-effects meta-analysis and a meta-regression of drug resistance by publication year. Between-study heterogeneity was assessed using the I^2^ test, which is defined as the percentage of total variation across studies that is due to heterogeneity rather than chance.^17^ We created funnel plots and performed Egger's test to assess potential publication bias. Multiple imputations were used to deal with missing data.^18^ Mixed-effects logistic regression analyses were performed to evaluate potential risk factors for drug resistance. First, all predictors with p < 0·2 were taken forward to a multivariable model. The model was reduced by backward selection of the least contributing variables, based on the minimisation of the Akaike information criterion. Internal-external cross-validation was performed by leaving one cohort out of the training dataset and validating the model on each holdout cohort. The area under the receiver operating curve (AUC) was computed by merging the predictions of each cross-validation iteration.^19^ As sensitivity analyses, we assessed the ability to predict freedom of any seizure and freedom of GTCS in the last one, two and five years of follow-up, based on the same predictors.

Cox proportional hazards analyses were performed to assess the time to seizure recurrence after start of ASM withdrawal. Univariable predictors at p < 0·2 were used for multivariable analyses, after which backward selection was performed to remove the least contributing predictors. We performed an internal-external validation by splitting the 18 cohorts with data on post-withdrawal seizure recurrence into three datasets of 6 cohorts, balanced on sample size. We trained the prediction model on 12/18 cohorts and assessed the external predictive value of this model on the left-out 6 cohorts, quantified with the concordance-statistic (C-statistic).^20^ Such non-random internal-external validations qualify as external validations of the model.^21^^,^^22^

AUC and C-statistic values range between 0 and 1, where a value of 0.5 represents no better prediction than chance, and 1 represents perfect predictive performance. A value < 0.7 is generally considered poor, ≥0.7 is deemed acceptable, and ≥0.8 is considered excellent.^23^

All statistical analyses were performed in RStudio Version 1.3.1093, using the packages: MICE, metafor, glmer, rms, coxme, rsample, purrr, survminer, tidyverse, ggplot, and survAUC.

### Nomogram and web-based risk assessment tool

To aid use in clinical practice, we converted our multivariable models to nomograms and web-based tools. The nomograms are visual representations of the mixed-effects logistic regression analysis on drug resistance and the Cox proportional hazards model on seizure recurrence within 2 and 5 years. They come with instructions to manually estimate clinical outcomes for an individual. Similarly, we translated the models into web-based tools where a user can fill in predictors to obtain the associated probability of a clinical outcome for an individual.

### Role of the funding source

The funder had no role in the study design, data collection, data analysis, data interpretation, drafting of the report or the decision to submit. RS, DA-T, WMO, BPCK, FEJ, KPJB had full access to all the data. All authors interpreted results, reviewed and critically revised the article, and approved the final version for submission.

## Results

**Fig. 1:**
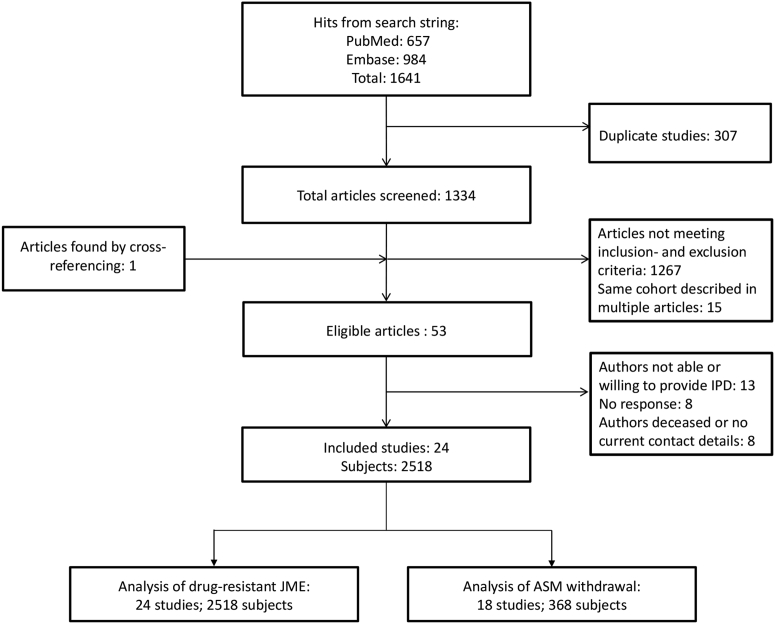
Flowchart of search strategy and study selection.s

We screened 1334 articles and identified 53 eligible studies (Fig. 1). The authors of 24 of these studies were able and willing to provide IPD. Four were prospective, 19 were retrospective, and one study had a mixed retrospective and prospective design (see Supplementary Table S2 for study characteristics).^24^, ^25^, ^26^, ^27^, ^28^, ^29^, ^30^, ^31^, ^32^, ^33^, ^34^, ^35^, ^36^, ^37^, ^38^, ^39^, ^40^, ^41^, ^42^, ^43^, ^44^ Eligible articles of which IPD was not included were similar in design and proportion of drug resistance, although some were markedly older and smaller (Supplementary Table S3). Meta-regression incorporating non-included articles, did not show changes in drug resistance by publication year (p = 0.44; Supplementary Fig. S1). Based on the Newcastle Ottowa assessment scale, the original publications' quality scores ranged between three to seven (mean 4·4; Supplementary Table S4).

In total, 2518 individuals from 18 countries and various ethnicities were included in the predictive analyses of drug resistance. Missing data before imputation ranged between 0% and 38% per variable (median 10·0%, IQR 0–19·4%). Among variables included in the drug resistance analysis, three variables had missing data between 25% and 40%; eight variables were missing between 10% and 25%, five variables between 1% and 10%, and data was complete for seven variables (Supplementary Fig. S2). Among variables included in the seizure recurrence analysis, two variables were missing between 25 and 45%, nine variables between 10% and 25%, ten variables between 1% and 10%, and data were complete for ten variables (Supplementary Fig. S3).

Follow-up duration ranged from one to 73 years (median 8·0, IQR 4·0–16·0). Information on current ASM treatment was available for 2365 people, of which 805 (34%) were on multiple ASMs, and 1560 (66%) were on monotherapy (Supplementary Table S2). Among those on monotherapy, valproate was most often used (n = 826, 54%), followed by levetiracetam (n = 352, 23%) and lamotrigine (n = 154, 10%). Amongst 2216 people with known past ASM treatment, 661 (30%) were still taking the first prescribed medication, 727 (33%) had used one, and 828 (37%) used two or more previous ASMs (Supplementary Table S6). A subset of 368 people with JME (15% of the total cohort) had attempted to withdraw from ASM treatment at any time during follow-up (median follow-up after the start of withdrawal: 4·0 years, IQR 1·5–9·0). Of these, 112 (30%) were not using any ASM at the last follow-up.

Meta-analysis showed that 29% (95%CI 23–36%) of people with JME were drug-resistant (Supplementary Fig. S4), with significant heterogeneity between studies (I^2^ = 88%, p < 0·0001). Funnel plots (Supplementary Fig. S4) and Egger's test (p = 0.46) did not show evidence for publication bias. Amongst 388 drug-resistant people with data on the most extended period of seizure freedom, 250 (64%) had never been seizure-free for more than 12 months, and 58 (15%) were never free of seizures for more than 1 month at any point.

**Fig. 2:**
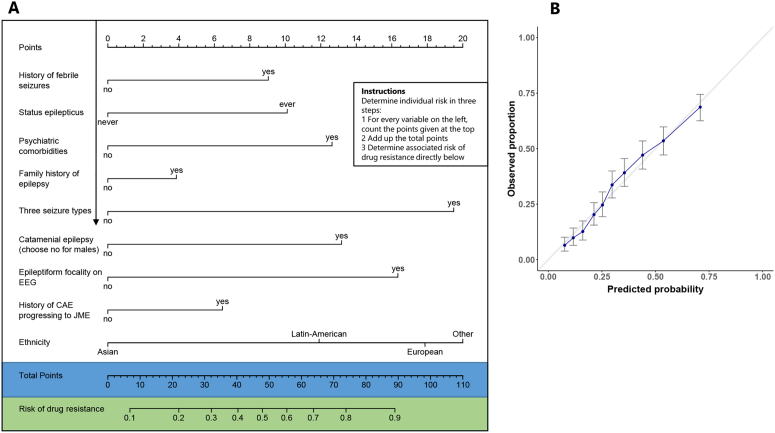
**(A)** Nomogram for prediction of drug-resistant JME. For example, a Caucasian (18 points) girl with a history of febrile seizures (9), who never had a status epilepticus, who has a psychiatric comorbidity (12.5), no family history of epilepsy (0), three seizure types (19.5), catamenial epilepsy (13), focal epileptiform activity on EEG (17), no history of childhood absence epilepsy (CAE; 0), has a total 89 points, corresponding to a 90% risk of drug resistance. **(B)** Calibration plot comparing observed and predicted probabilities, which should ideally follow the diagonal line.

Univariable mixed-effects logistic regression analysis identified 18 predictors of drug resistance at p < 0·2 (Table 1; distributions of drug resistance and seizure recurrence concerning potential predictors are in Supplementary Table S7 ), some of which were correlated (see Supplementary Table S7). After backward selection in multivariable analyses, we identified nine variables with independent predictive values for drug-resistant JME (Fig. 2, Supplemental Table S9): psychiatric comorbidities, three seizure types, focal epileptiform activity on EEG, catamenial epilepsy, status epilepticus, history of febrile seizures, family history of epilepsy, history of CAE progressing to JME, and ethnicity. Associations were similar when restricted to 1163 cases with complete data (Supplementary Table S10).

**Table 1:** Univariate predictors of drug resistance and seizure recurrence after ASM withdrawal.

Predictor	n (%) or median (IQR)	Association with drug resistance		Association with seizure recurrence after ASM withdrawal	
		OR (95% CI)	p-value	HR (95% CI)	p-value
Gender					
Male	978/2518 (38.8%)	0.84 (0.69–1.03)	0.088	0.92 (0.71–1.20)	0.55
Female	1540/2518 (61.2%)	Ref	–	Ref	–
Age at first seizure (years)	15 (12–17)	0.96 (0.94–0.98)	0.00013	1.00 (0.97–1.02)	0.79
Age at last moment of follow-up (years)	29 (23–38)	1.01 (0.76–1.36)	0.922	0.38 (0.27–0.55)	<0.0001
Diagnostic delay (months)	1 (0–3)	1.13 (0.97–1.32)	0.12	0.99 (0.91–1.07)	0.82
Ethnicity					
Caucasian	1263/2054 (61.5%)	Ref	–	Ref	–
Asian	510/2054 (24.8%)	0.46 (0.24–0.88)	0.022	0.91 (0.68–1.22)	0.53
Latin-American	85/2054 (4.2%)	1.31 (0.52–3.31)	0.56	0.94 (0.45–1.96)	0.87
Other or admixed	196/2054 (9.5%)	1.01 (0.32–3.20)	0.98	0.82 (0.26–2.61)	0.74
History of febrile seizures					
Yes	205/2331 (8.8%)	1.57 (1.14–2.17)	0.0065	0.69 (0.42–1.13)	0.14
No	2126/2331 (91.2%)	Ref	–	Ref	–
Ever experienced status epilepticus					
Yes	68/2124 (3.2%)	2.29 (1.37–3.84)	0.0018	1.22 (0.50–2.97)	0.66
No	2056/2124 (96.8%)	Ref	–	Ref	
Developmental delay					
Yes	20/2011 (1%)	1.85 (0.76–4.49)	0.18		0.30
No	1991/2011 (99.0%)	Ref	–	1.83 (0.58–5.74)	
Neurological comorbidities					
Yes	190/2518 (13.5%)	1.19 (0.84–1.68)	0.33	1.07 (0.76–1.51)	0.68
No	2328/2518 (86.5%)	Ref	–		–
Psychiatric comorbidities					
Yes	416/2018 (20.6%)	2.27 (1.78–2.89)	<0.0001	1.28 (0.95–1.74)	0.11
No	1602/2018 (79.4%)	Ref	–	Ref	–
Family history of epilepsy					
Yes	796/2318 (34.3%)	1.22 (0.99–1.51)	0.068	0.90 (0.68–1.19)	0.45
No	1522/2318 (65.7%)	Ref	–	Ref	–
Myoclonic seizures					
Yes	2481/2518 (98.5%)	Ref	–	0.37 (0.02–5.92)	0.49
No	37/2518 (1.5%)	0.95 (0.41–2.18)	0.90	Ref	–
Generalised tonic-clonic seizures (GTCS)					
Yes	2322/2518 (92.2%)	1.42 (0.90–2.26)	0.14	1.20 (0.71–2.03)	0.49
No	196/2518 (7.8%)	Ref	–	Ref	–
Absence seizures					
Yes	788/2518 (31.3%)	2.93 (2.36–3.63)	<0.0001	1.48 (1.14–1.91)	0.0031
No	1730/2518 (68.7%)	Ref	–	Ref	–
Three seizure types					
Yes	748/2518 (29.7%)	3.26 (2.64–4.02)	<0.0001	1.43 (1.10–1.86)	0.0089
No	1770/2518 (70.3%)	Ref	–	Ref	–
History of childhood absence epilepsy (CAE) progressing to JME					
Yes	193/2247 (8.6%)	2.34 (1.67–3.29)	<0.0001	0.89 (0.56–1.41)	0.61
No	2054/2247 (91.4%)	Ref	–	Ref	–
Praxis-induced seizures					
Yes	103/1609 (6.4%)	1.76 (1.05–2.95)	0.034	1.10 (0.61–1.99)	0.75
No	1506/1609 (93.6%)	Ref	–	Ref	–
Epileptiform focality on EEG					
Yes	325/2042 (16%)	2.23 (1.59–3.13)	<0.0001	1.19 (0.86–1.66)	0.29
No	1717/2042 (84.0%)	Ref	–	Ref	–
Photoparoxysmal response					
Yes	485/2266 (21.4%)	1.26 (0.99–1.61)	0.059	1.28 (0.96–1.70)	0.096
No	1781/2266 (78.6%)	Ref	–	Ref	–
Motor seizures during sleep					
Yes	266/1563 (17%)	1.71 (1.21–2.41)	0.0032	0.79 (0.54–1.17)	0.25
No	1297/1563 (83.0%)	Ref	–	Ref	–
Catamenial epilepsy^a^					
Yes	156/915 (17.0%)	2.13 (1.44–3.16)	<0.0001	1.27 (0.82–1.98)	0.28
No	759/915 (83.0%)	Ref	–	Ref	–
Age at start of ASM reduction (years)	24 (19–31.75)	–	–	0.96 (0.95–0.98)	<0.0001
Epilepsy duration before remission (years)	8 (3–14)	–	–	0.97 (0.95–0.98)	<0.0001
Seizure-free interval before start of ASM reduction (years)	3 (2–5)	–	–	0.58 (0.46–0.74)	<0.0001
Number of GTCS before remission					
<10	238/289 (82.7%)	–	–	Ref	–
≥10	51/289 (17.3%)			1.03 (0.69–1.53)	0.90
EEG abnormality before reduction of ASM					
Yes	67/221 (30.0%)	–	–	0.93 (0.65–1.34)	0.70
No	154/221 (70.0%)			Ref	–
Number of ASMs used at start of reduction	1 (1–1)	–	–	1.36 (1.01–1.82)	0.045

The first column notes the prevalence (%) for categorical variables or the median (IQR) for numerical variables. Missing data differs per variable, and proportions are calculated based on non-missing data. Odds ratios (OR) are computed for the association with drug resistance and hazard ratios (HR) are computed to assess associations with seizure recurrence. Positive HR or OR values for numerical variables represent increased risk associated with a higher value. The last six variables are specific for subjects that have attempted ASM withdrawal thus we did not calculate associations with drug resistance.

a Catamenial epilepsy is a female-specific risk factor. Thus, we have calculated the proportion as a fraction of female subjects. ASM: anti-seizure medication.

We performed internal-external cross-validation to assess the external predictive value of the multivariable model, which showed an AUC of 0·70 (95%CI 0·67–0·72). The AUC varied between 0·56 and 0·84 per left-out cohort (median 0·70, IQR 0·66–0·76), with smaller cohorts on both ends of the distribution (Supplementary Table S11). A plot of predicted against observed probabilities showed excellent calibration (Fig. 2B).

As further sensitivity analyses, we assessed how well we could predict freedom of any seizure and freedom of GTCS in the last one, two and five years of follow-up. We used the same predictors (see Supplementary Table S12 for a correlation matrix of outcome measures), without considering the pre-treatment seizure interval and the number and appropriateness of each drug trial as in the drug resistance analyses. The AUC for freedom of any seizure was 0·67 (95%CI 0·65–0·69) for the last year, 0·63 (0·61–0·66) for two years, and 0·59 (0·56–0·61) for five years. The AUC of the prediction model for freedom of GTCS, was 0·64 (0·61–0·67) for the last year, 0·62 (0·59–0·64) for two years and 0·63 (0·60–0·66) for five years.

**Fig. 3:**
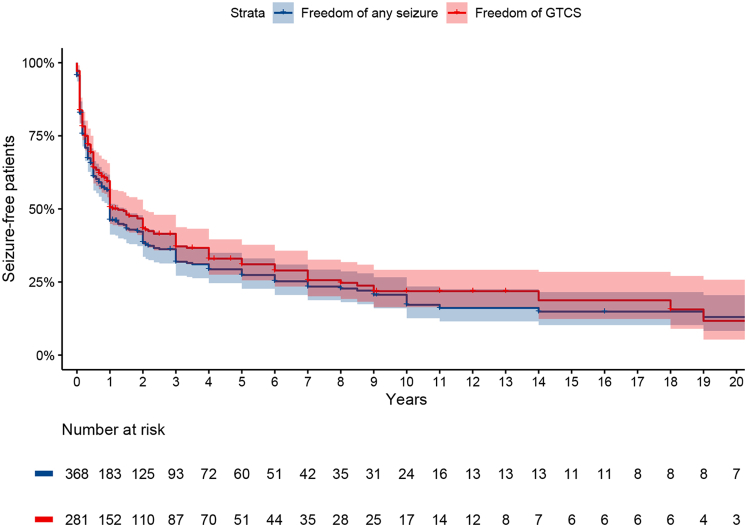
**Survival curve for seizure-freedom after initiation of ASM withdrawal.** Freedom of any seizure after withdrawal (blue) and freedom of generalised tonic-clonic seizures (GTCS; red) after withdrawal are displayed, with the 95% confidence interval in shaded colours. The X-axis represents the years after start of withdrawal. The number of individuals at risk is displayed below risk for each time point.

We performed survival analyses to assess the recurrence of seizures in people who attempted to withdraw their ASM treatment (n = 368). These individuals were older and included more people of Asian ethnicity compared to people who did not try to withdraw treatment but did not differ in other predictors of drug resistance (Supplementary Table S13). Five years after initiation of ASM withdrawal, 73% (95%CI 67–78%) had experienced seizure relapses (Fig. 3). Slightly fewer (69%, 95%CI 62–74%) had a seizure recurrence when assessing only GTCS. Amongst 116 people restarting treatment after a seizure recurrence and followed at least two years after recurrence, 90 (78%) regained freedom of any seizure for at least 12 months at the last follow-up.

**Fig. 4:**
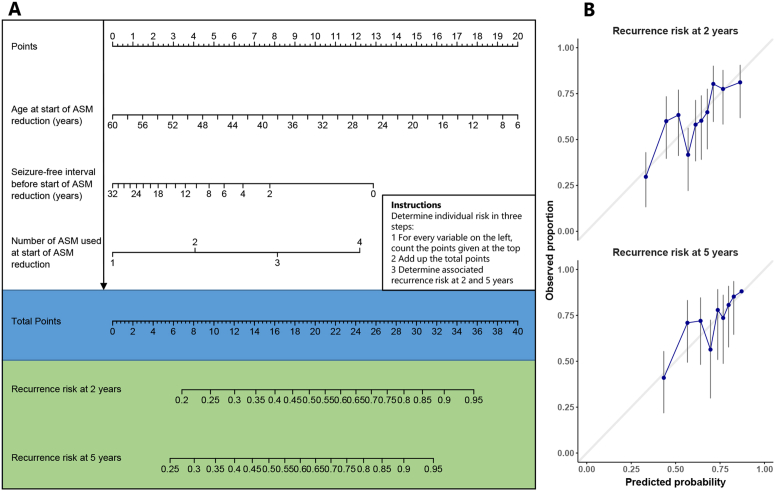
**(A)** Nomogram for predicting recurrence of any seizure after ASM withdrawal in people with JME. For example, someone with JME who is 44 years old at initiating withdrawal (6 points), who has been seizure-free for the last four years (6 points) and is currently using 1 ASM (0 points) has a total score of 12, which corresponds to a 37% chance of recurrence in 2 years and a 48% chance of recurrence at five years. **(B)** Calibration plot comparing observed and predicted probabilities, which should ideally follow the diagonal line.

Univariable analyses showed ten predictors of seizure recurrence at p < 0·2 (Table 1). Subsequent multivariable analyses and backwards selection showed three variables with independent predictive value (Supplementary Table S14, Fig. 4): age at withdrawal, the seizure-free interval before withdrawal and number of ASM used at the start of reduction. Restricting analyses to 282 complete cases did not affect these associations (Supplementary Table S15). Internal-external cross-validations by creating three splits of our data (6 cohorts per split) showed similar external predictive performance for all three data splits (split 1: n = 119, C-statistic = 0·68; split 2: n = 121, C-statistic = 0·74; split 3: n = 128, C-statistic = 0·70), with an average C-statistic of 0·70 (95%CI 0·68–0·73). Plotting observed against predicted probabilities showed good calibration (Fig. 4B). As an example, only 44% (95%CI 33–53%) of people older than 30 years at withdrawal had a recurrence of seizures within two years, compared to 68% (95%CI 61–73%) of those less than 30 years old (Supplementary Fig. S5). Assessment of recurrence of GTCS after ASM withdrawal revealed one additional risk factor: people who had more than ten GTCS before remission of seizures (Supplementary Table S16), but the external predictive value for recurrence of GTCS was poor (split 1: n = 94, C-statistic = 0·64; split 2: n = 94, C-statistic = 0·56; split 3: n = 93, C-statistic = 0·61), with an average C-statistic of 0·61 (95%CI 0·58–0·63).

## Discussion

We collected IPD from a large group of people, enabling the creation of prediction models for individual assessment of drug resistance (n = 2518) and seizure recurrence risk after withdrawal of ASM treatment (n = 368) in JME. We validated the prediction models and found good calibration. Three-quarters of people who attempted to withdraw ASMs experienced a seizure recurrence within five years, for which we found three predictors. A third of people with JME were drug-resistant, for which we found nine independent predictors.

We confirmed multiple previously found risk factors of drug resistance.^4^^,^^10^^,^^45^^,^^46^ Similar to a previous study investigating sex-specific risk factors, we found catamenial epilepsy and absence seizures strongly associated with drug-resistant JME.^46^ To maximise statistical power for our prediction model, we did not perform sex-stratified analyses. Therefore, we cannot confirm earlier reported female-specific risk factors of drug-resistant JME.^46^ Some of these predictors were correlated, and we included all possible predictors in a sufficiently powered single model. We based our multivariable prediction model on routinely available variables for the model to be freely used in clinical practice. Prediction of a relatively high risk of drug resistance could have implications for counselling and treatment guidance. For example, a Caucasian woman with catamenial epilepsy, three seizure types and psychiatric comorbidities has a high risk of drug resistance. Early referral to a specialised epilepsy clinic should then be considered. Valproate may be regarded as an option at childbearing age, but only after careful consideration of the superior efficacy versus teratogenicity.^47^ When considering to start add-on therapy, the attending physician should base the choice of add-on ASM on the predominant seizure type.^48^

We identified two predictors not previously associated with drug-resistant JME: family history of epilepsy as a risk factor, and Asian ethnicity was protective compared to Caucasians. The most likely explanation for this association is that both predictors are proxies of the presumed population-specific genetic basis of JME.^49^ Alternatively, there might be differences in under-reporting seizures relating to cultural and ethnic differences, social stigma, or driving licence regulations.^50^^,^^51^ JME, like other genetic generalised epilepsy syndromes, is highly polygenic with several thousand common genetic variants explaining 62% of JME liability, much of which is shared between epilepsy subtypes.^49^^,^^52^ A family history of disease is associated with an increased polygenic disease burden,^53^ which might result in a more severe phenotype with increased risk of drug resistance.

We found three predictors of seizure recurrence after ASM withdrawal. There are several predictors of seizure recurrence in the broader epilepsy population,^11^ but it is unknown whether this could be generalised to specific syndromes such as JME. We found that only one out of eight previously identified risk factors for seizure recurrence was predictive in JME. Interestingly, the strongest predictor in JME, age at ASM withdrawal, has an opposite direction of effect than in the broader epilepsy population^11^: older age at withdrawal reduces the risk of seizure recurrence in JME. In contrast, it increases seizure recurrence risk in a large population of other epilepsy forms.^11^ These findings underscore the benefit of assessing a specific epilepsy syndrome instead of pooling heterogeneous epilepsy subtypes. We found that two-thirds of people with JME had a recurrence of seizures within five years, much higher than for other types of epilepsy.^11^ This recurrence risk aligns with the common perception that people with JME require lifelong treatment. A subset of people, particularly older people using one ASM with prolonged seizure freedom, may have a good chance of remaining seizure-free. This is in line with the finding that myoclonic seizures often cease in the fourth decade.^26^

Our results showed a higher AUC for drug resistance prediction than seizure freedom prediction in the last one, two, and five years. The higher AUC suggests that the formal definition of drug resistance as defined by the ILAE,^16^ which considers the pre-treatment seizure interval and the number and appropriateness of each drug trial, is a more robust outcome measure than seizure freedom alone. We did not achieve excellent predictive accuracy, despite the large sample size and the checking of various independent predictors. One explanation could be that drug response may change over time, whereas the predictors remain stable throughout life.^54^ Indeed, repeated remissions and relapses are common in epilepsy (although not explicitly assessed in JME-only cohorts),^55^ and some people resistant to the first two ASM regimens become seizure-free upon a third or later regimen.^56^^,^^57^ Conversely, two-thirds of people with drug resistance had a prior episode of remission longer than one year.^58^ The ILAE definition of drug resistance outperforms previous definitions but there remains a substantial inter- and intra-observer variability.^59^

We found a higher AUC for predicting seizure freedom and recurrence of seizures after ASM withdrawal when assessing any seizure type compared to the analyses confined to GTCS. Analyses on GTCS may lack statistical power. Alternatively, freedom of GTCS might be inherently more challenging to predict since GTCS often occur less frequently than myoclonic or absence seizures. For example, assessing GTCS freedom in the last year of follow-up might be an unreliable measure for someone only having GTCS every other year. Hence, we would advocate using the ILAE definition of drug resistance, which considers all seizure types and pre-treatment seizure intervals.

Our study has limitations. The included cohorts were primarily obtained from tertiary care centres, potentially limiting the generalisability of our prediction model to primary and secondary care. Potential selection bias and selective loss to follow-up of drug-responsive people could further reduce the representativeness of our dataset. We provided a standardised data entry sheet, but significant intra- and inter-observer variability likely remain. We found considerable heterogeneity between cohorts. Potential sources of heterogeneity include differences in demography, study ascertainment, country-specific healthcare organisation and accessibility. In particular, differences in ethnicity between studies could explain part of the heterogeneity in the proportion of drug resistance, and age differences might explain heterogeneity in seizure recurrence rate after withdrawal. We mitigated the influence of between-study heterogeneity by using random-effect statistical models, although heterogeneity might have still limited the predictive performance of our models. As most studies were several years old, collecting all potential predictors for each individuals was impossible. We mitigated this by performing multiple imputations of missing data, reducing bias and increasing precision.^60^ We included only readily available clinical predictors. It is possible that the predictive performance could be improved if other variables such as genetic diagnostic investigations, advanced EEG analysis, and functional MRI measures were included. Only six ethnic Asians were included in studies outside of Asia, and no Caucasians were included in Asian studies. Therefore, we were unable to perform stratified analyses on ethnicity by location JME may be associated with elevated impulsivity, potentially due to disruption of cortico-striatal and thalamocortical networks. This impulsivity may lead to challenging lifestyles and issues in treatment adherence.^61^ We attempted to mitigate the influence of lifestyle and treatment adherence by excluding people with pseudo-resistance. We could not collect data on trait impulsivity, so we cannot assess its potential association with drug resistance. Lastly, the small proportion of individuals that attempted to withdraw treatment limited our analyses on predictors of seizure recurrence. We were unable to find an individually large enough cohort to perform external validation. However, our internal-external cross-validations performed by creating three splits of the 18 cohorts showed robust external predictive performance of our models,^21^^,^^22^ suggesting that the predictors are similar across different populations. It is essential to consider these limitations in the context of the evidence before this study. Knowledge on JME prognosis and risk factors of drug resistance is currently based on single-centre cohort studies without validation, ASM withdrawal is rarely attempted, and there are currently no known predictors to guide a safe attempt. Despite some unavoidable limitations, a meta-analysis of IPD represents the best available evidence at this moment.^62^

In conclusion, we assessed whether we could predict the likelihood that an individual with JME will become drug-resistant or has a seizure recurrence after ASM withdrawal. After validating these predictions, we created nomograms and developed publicly accessible web-based tools to help estimate individualised risks (http://epilepsypredictiontools.info/). We expect that the models will aid in improving and personalising the treatment and counselling of people with JME.

## Contributors

RS, FEJ, HJL, JWS, BPCK, WMO and KPJB contributed to study conceptualisation and design. RS and DA-T analysed the data and created the figures. WMO and HJL supervised the statistical analyses. RS and DA-T verified the integrity of the full dataset. RS, DA-T, WMO, BPCK, FEJ, KPJB have full access to all the data. RS wrote the first draft of the report, with input from DA-T, WMO, FEJ, BPCK and KPJ. Data were obtained by AAA, MF, GC, SJ, AP, ÇÖ, SA, JG, CPB, LJS, MJB, GU, AR, JH, ET, RK, ECI, CDB, JPS, LEH-V, MLM-A,YZ, DZ, NP, NS, GJ, SB, MJ, PK, MS, KKS, BJV, MH, LGV, SS, BB, EAA, FvP, JS, US, AV-A, IK, WD, JWS. All authors interpreted results, reviewed and critically revised the article, and approved the final version for submission.

## Data sharing statement

The individualised prediction models for drug resistance and recurrence of seizures after ASM withdrawal in JME are available as nomograms in this manuscript. They will be made available upon publication as a web-based tool at http://epilepsypredictiontools.info/. The main analyses’ study protocol and R scripts are available on https://osf.io/b9zjc/. The signed data-sharing agreements between the different cohorts participating in this study do not allow the de-identified individual participant dataset to be publicly released. An exception can be made to replicate the results in this manuscript by an academic third party, after signing data sharing agreements with all collaborating centres.

## Declaration of interests

AAA received a grant from the 10.13039/501100012155National Institute for Medical Research Development, royalties for a book publication from 10.13039/501100007723Oxford University Press, and speaker fees from Cobel Daruo, Tekaje, and Raymand Rad. CPB received research grants and honoraria from 10.13039/100011110UCB and 10.13039/501100003769Eisai, support for attending meetings by UCB, and served in the advisory board of Arvelle. CDB received consulting fees and honoraria from 10.13039/100014114GW Pharmaceuticals, 10.13039/100015661UCB Pharma, 10.13039/501100003769Eisai, 10.13039/501100006546Angelini Pharma and Bial. JPS received grants from the 10.13039/100000002National Institutes of Health, 10.13039/100000005Department of Defense, and the 10.13039/100000001National Science Foundation; and consulting fees from 10.13039/100015661UCB Pharma, AdCel Biopharma, LLC, iFovea, SK Life Sciences, and 10.13039/100013410LivaNova; and has stock options for iFovea and AdCel Biopharma. LEH-V participates in the Young Epilepsy Society, received speaker honorario from Armstrong, and was supported by Abbott pharmaceuticals to attend the Mexican Congress of Neurology. NP received honoraria from Zogenix and Ethos for Angelini Pharma. NS received honoraria from Biomarin, Livanova, GW Pharma, Zogenix and Marinus; and support to attend meetings from Livanova, GW Pharma and Zogenix; and participated on a data safety monitoring board for Marinus. SB received speaker fees from Eisai. PK received lecture honorarium from UCB Pharma and Eisai, consulting fees from 10.13039/501100003769Eisai and 10.13039/100013410LivaNova and his institution received research grants from 10.13039/100015661UCB Pharma and 10.13039/501100003769Eisai. MS received speaker honoraria from UCB Pharma and Eisai. KKS received research grants from the 10.13039/501100005416Norwegian Research Council, the DAMFoundation and the Norwegian National Advisory Unit on Rare diseases; and a networking grant from the 10.13039/501100004785NordForsk Foundation; and she acted as a paid PhD defense opponent at the University of Bergen, and attended a meeting for Nordic clinicians organised by Eisai. BJV received grants from the 10.13039/501100007870German Society for Epileptology and the 10.13039/501100001659Deutsche Forschungsgemeinschaft; and honoraria from University Medical Center Schleswig–Holstein and Cornelsen Verlag. MH received consulting fees and honoraria from Arvelle, Bial, Desitin, Eisai, GW Pharmaceuticals, UCB Pharma, and Zogenix. FvP has received speaker honoraria from Bial, Eisai, GW Pharmaceutical companies, Angelinipharma, Zogenix and UCB Pharma; and scientific advisory board honoraria from GW Pharmaceutical companies, UCB Pharma, and Angelinipharma. WD's salary is part-funded by The University of Melbourne; he has received travel, investigator-initiated, scientific advisory board and speaker honoraria from UCB Pharma Australia and Global; investigator-initiated, scientific advisory board, travel and speaker honoraria from Eisai Australia and Global; advisory board honoraria from Liva Nova and Tilray; educational grants from Novartis Pharmaceuticals, 10.13039/100014476Pfizer Pharmaceuticals and Sanofi-Synthelabo; educational; travel and fellowship grants from GSK Neurology Australia, and honoraria from SciGen Pharmaceuticals; and he has an equity interest in the device company EpiMinder. CPB received honaries and research support from EISAI, UCB and Arvelle. ET received speaker's honoraria from Arvelle, Abbott, Angelini Pharma, UCB, Biogen, Gerot-Lannacher, Bial, Eisai, Epilog, Takeda, Newbridge, Hikma, GW Pharmaceuticals, Sunovion Pharmaceuticals Inc., LivaNova and Novartis; consultancy funds from 10.13039/501100006546Angelini Pharma, Argenix, Arvelle, Epilog, 10.13039/100011110UCB, 10.13039/100005614Biogen, Gerot-Lannach, Bial, 10.13039/501100003769Eisai, 10.13039/100007723Takeda, Newbridge, 10.13039/100014114GW Pharmaceuticals, 10.13039/100009655Sunovion Pharmaceuticals Inc., Marinus, and 10.13039/100004336Novartis; directorship funds from Neuroconsult GmbH. ET's Institution received grants from 10.13039/100005614Biogen, Red Bull, 10.13039/100004334Merck, 10.13039/100011110UCB, 10.13039/501100000780European Union, FWF Österreichischer Fond zur Wissenschaftsförderung, and 10.13039/501100007148Bundesministerium für Wissenschaft und Forschung. JWS reports personal fees from Arvelle, personal fees from 10.13039/100011110UCB, grants from UCB, grants from 10.13039/100003997NEF, grants from UCB, personal fees from 10.13039/100012739Zogenix, grants from GW Phama, outside the submitted work; and his current position is endowed by the Epilepsy Society, he is a member of the Editorial Board of the Lancet Neurology, and receives research support from the Marvin Weil Epilepsy Research Fund. All other authors declare no potential competing interests. None of the above mentioned declarations represent a conflict of interest directly related to the present publication.
