# Supplementary material for: Individualised prediction of drug resistance and seizure recurrence after medication withdrawal in people with juvenile myoclonic epilepsy: A systematic review and individual participant data meta-analysis
Source: eClinicalMedicine. 2022 Nov 11;53:101732. doi: 10.1016/j.eclinm.2022.101732 (PMC9716332; doi:10.1016/j.eclinm.2022.101732)
Supplement: Supplementary Figures S1–S5, Tables S1–S16, and Supplementary Methods [file mmc1.pdf]

**Supplementary figures and tables with the manuscript: “Individualised prediction of drug resistance and seizure recurrence after medication withdrawal in people with juvenile myoclonic epilepsy: a systematic review and individual participant data meta-analysis”**

**List of EpiPGX members**

Andreja Avbersek, Costin Leu, Kristin Heggeli, Rita Demurtas, Joseph Willis, Douglas Speed, Narek Sargsyan, Krishna Chinthapalli, Antonietta Coppola, Antonio Gambardella, Stefan Wolking, Felicitas Becker, Sarah Rau, Christian Hengsbach, Yvonne G. Weber, Bianca Berghuis, Wolfram S. Kunz, Mark McCormack, Norman Delanty, Ellen Campbell, Lárus J. Gudmundsson, Andres Ingason, Kári Stefánsson, Reinhard Schneider, Rudi Balling, Pauls Auce, Ben Francis, Andrea Jorgensen, Andrew Morris, Sarah Langley, Prashant Srivastava, Martin Brodie, Marian Todaro, Slave Petrovski, Jane Hutton, Fritz Zimprich, Martin Krenn, Hiltrud Muhle, Karl Martin Klein, Rikke Moller, Marina Nikanorova, Sarah Weckhuysen, Zvonka Rener-Primec, Gianpiero L. Cavalleri, John Craig, Chantal Depondt, Michael R. Johnson, Bobby P. C. Koeleman, Roland Krause, Holger Lerche, Anthony G. Marson, Terence J. O'Brien, Josemir W. Sander, Graeme J. Sills, Hreinn Stefansson, Pasquale Striano, Federico Zara and Sanjay M. Sisodiya

| Variable | Definition/instructions for data entry |
|---|---|
| Duration of freedom from any seizure | Months between last seizure of any type (myoclonic, absence or generalised-tonic clonic seizures [GTCS]) and last moment of follow-up. E.g., input ten if the subject was last followed up 20 months ago and last had a myoclonic seizure 30 months ago. |
| Duration of freedom from generalised tonic-clonic seizures | Months between last tonic-clonic seizure and last moment of follow-up. E.g., input 40 if the subject was last followed up 20 months ago and last had a tonic-clonic seizure 60 months ago. |
| Longest period ever of freedom from any seizure | Input the longest period of seizure freedom at any moment of any seizure type. E.g., input 120 if a person has been seizure-free for ten years from age twenty to thirty, even though he/she last had a seizure one year before last follow-up. |
| Drug-resistant epilepsy according to ILAE definition (Kwan et al., 2010) | Input either yes or no according to: https://pubmed.ncbi.nlm.nih.gov/19889013/.<br>Do not include 'pseudo-refractory' subjects for this study (i.e. people with seizures due to non-compliance, inadequate treatment or abnormal lifestyle). |
| Sex | Input biological sex at birth ('male' or 'female'). |
| Age at first seizure | Age (in years) at which the first suspected unprovoked seizure of any kind has occurred (febrile seizures not included). E.g., input six if the patient first had an absence seizure at six years old. Or input 12 if the patient first had a myoclonic seizure at 12 years old. |
| Age at last follow-up | Age (in years) at the moment of last follow-up of which treatment outcome details are available. E.g., input 35 if the patient was last seen at 35 years of age. |
| Total duration of follow-up | Total number of years of which clinical data is available for the subject. E.g., input eight if a patient had a first seizure at 12 years old, and clinical data is available from age 27, while he is now 35 years old. |
| Diagnostic delay | Years between first suspected seizure and diagnosis of epilepsy. E.g., input three if the patient had a first seizure at age twelve but was diagnosed with epilepsy at 15 years of age. |
| Country of study | Country where the study was conducted. If the study was multi-centre between different countries, input the country where the subject was under treatment. |
| Ethnicity | Input the subject's ethnicity in any of the following categories: Caucasian, Asian, African, Latin-American, other or admixed. |
| History of febrile seizures | Input yes if the subject has ever experienced febrile seizures. Input no if the subject has never experienced febrile seizures. |
| Status epilepticus | Input 'ever' if the subject has ever experienced status epilepticus (SE). Input 'never' if the subject has never experienced SE. SE is defined as a generalised tonic-clonic seizure >5min, an absence seizure >15 min and/or a cluster of myoclonic seizures lasting >30 min. |
| ASM's used at last follow-up | Input all ASM's the subject uses at the last moment of follow-up. |
| Previously used ASM's | Input all ASM's the subject has ever tried in the past. |
| Developmental delay (IQ or DQ <70) | Input yes if the subject has an intelligence quotient (IQ) or developmental quotient (DQ) lower than 70, or if there is a very strong suspicion of such a delay, which has never been formally tested. Input no if no developmental delay is suspected. |
| Neurological comorbidities | Free text. Please input any neurological comorbidity other than epilepsy with which the patient has ever been diagnosed. Leave blank if the patient has no neurological comorbidities. |
| Psychiatric comorbidities | Input yes if the patient has ever received a DSM-classified psychiatric diagnosis. Input no if the patient has never received a psychiatric diagnosis. Leave blank if this information is unavailable. |
| Family history of epilepsy 1st or 2nd degree | Input yes if the patient has a first degree (parents or sibling) or second degree (grandparents, uncles, aunts, first cousins) relative with a diagnosis of epilepsy. If possible, try to exclude familial cases of symptomatic or lesional epilepsy. |
| Absence seizures | Input yes if the subject has ever experienced one or more absence seizures (as judged by the treating physician). |
| Generalized tonic-clonic seizures (GTCS) | Input yes if the subject has ever experienced one or more generalised tonic-clonic seizures. |
| Myoclonic seizures | Input yes if the subject has ever experienced one or more myoclonic seizures. |

| | |
|---|---|
| Three seizure types (myoclonic, absence and GTCS) | Input yes if the subject has ever experienced all three seizures types associated with JME: myoclonic seizures, absence seizures and generalised tonic-clonic seizures. |
| History of childhood absence epilepsy (CAE) progressing to JME | Input yes if the patient was first diagnosed with CAE, after which he/she developed JME at a later age. Input no if the patient was never diagnosed with CAE. |
| Praxis-induced seizures | Input yes if the patient has seizures induced by praxis; i.e., if seizures are triggered by complex, cognition-guided tasks often involving visuomotor coordination and decision-making (for example, seizures provoked by calculation, playing chess or drawing. |
| Epileptiform focality on EEG | Input yes if focal epileptiform discharges have ever been detected on EEG. Input 'no' if no such features were visible. Focality is defined here as any unilateral epileptiform discharge. |
| Photoparoxysmal response | Input 'yes' if epileptiform discharges can be provoked by intermittent light stimulation during an EEG recording or if the patient experiences seizures provoked by photostimulation. |
| Motor seizures during sleep | Input yes if the patient has ever experienced motor seizures (e.g., GTCS) during sleep. Input no if the patient has never experienced such seizures. |
| Catamenial epilepsy (female only) | Input yes if a patient has catamenial epilepsy (defined as a change in seizure frequency with the menstrual cycle). Input no if the frequency does not change with the menstrual cycle. Leave blank if this is unknown. |
| ASM withdrawal attempted | Input yes if the patient has ever attempted to withdraw anti-seizure medication after a period of seizure freedom. Input no if the patient has never attempted this. If no, leave the following columns blank since these are not applicable. |
| Recurrence of any seizure after withdrawal | Input yes if the patient has had recurrence of any seizure type (myoclonic, absence or GTCS seizures) following ASM withdrawal. Input no if the patient was seizure-free at last follow-up. |
| If yes, time to recurrence of any seizure after start of ASM reduction (in months). | Input time in months after start of ASM reduction after which a seizure of any kind (absence, myoclonic or GTCS) has occurred. Leave blank if no seizure had recurred. E.g., input eight if the subject had a myoclonic seizure eight months after start of ASM reduction. |
| Recurrence of tonic-clonic seizures after withdrawal | Input yes if the patient has had recurrence of tonic-clonic seizures following ASM withdrawal. Input no if the patient had no tonic-clonic seizure or only other seizure types (myoclonic or absence) after ASM withdrawal. |
| If yes, time to recurrence of tonic-clonic seizures after start of ASM reduction (in months). | Input the time in months after the start of ASM reduction after a GTCS has occurred. Leave blank if no GTCS or only other seizure types have recurred after ASM withdrawal. E.g., input twenty if a GTCS has recurred 20 months after start of ASM reduction. |
| Age at start ASM reduction | Age in years at the moment of start of ASM reduction. E.g., input 30 if the subject started ASM reduction at age 30. |
| Epilepsy duration before remission | Input the interval between first and last seizure in whole years. E.g., input ten if the person had his/her first seizure at 12 years of age and the last seizure at 22 years of age, after which he/she remained seizure-free. |
| Seizure-free interval before start of ASM reduction | Input the interval between the last seizure and start of ASM reduction in years. E.g., input five if a person had a last seizure at age 22 and started ASM reduction at age 27. |
| Number of tonic-clonic seizures before remission | Input '0' if the subject has experienced less than ten tonic-clonic seizures before remission. Input '1' if the subject has experienced ten or more tonic-clonic seizures before remission. Leave blank if this is unknown. |
| EEG abnormality before ASM reduction | Input yes if there were any epileptiform abnormalities on the EEG before start of ASM reduction. Input no if there were no abnormalities. Leave blank if no EEG was performed before ASM reduction. |
| Number of ASMs used at moment of start ASM reduction | Input the number of ASM's that were used at the moment before start of ASM reduction. E.g., input one if the patient was only using valproic acid, or input two if the patient was using valproic acid and levetiracetam before start of ASM reduction. |
| Follow-up after start of ASM reduction | Input the time in months after start of ASM reduction and the last moment of follow-up. E.g., input 27 if the subject was last followed-up 27 months after start of ASM reduction. |

Supplementary Table 1: List of variables collected from each cohort, including the definitions sent to each collaborator with instructions for data entry.

| Study | Asadi-Pooya[1] | Baykan[2] | Cacao[3] | Cerulli Irelli[4] | Chowdhury[5] | EpiPGX[6] | Gesche[7] | Hernández-Vanegas[8] |
|---|---|---|---|---|---|---|---|---|
| Publication year | 2014 | 2013 | 2018 | 2020 | 2016 | 2019 | 2020 | 2016 |
| Study design | Retrospective | Retrospective and prospective | Retrospective | Retrospective | Retrospective | Retrospective | Retrospective | Retrospective |
| Country | Iran | Turkey | United Kingdom | Italy | Scotland | Multiple | Denmark | Mexico |
| Subjects | 180 | 38 | 239 | 116 | 83 | 145 | 165 | 85 |
| Male sex | 53/180 (29.4%) | 14/38 (36.8%) | 93/239 (38.9%) | 37/116 (31.9%) | 37/83 (44.6%) | 46/145 (31.7%) | 68/165 (41.2%) | 27/85 (31.8%) |
| Drug-resistant JME | 60/143 (42.0%) | 5/38 (13.2%) | 121/239 (50.6%) | 28/144 24.6%) | 32/83 (38.6%) | 34/145 (23.4%) | 22/147 (15.0%) | 29/82(35.4%) |
| Median age at first seizure (years) | 15 (13-16) | 14 (12-16) | 14 (12-16) | 14 (10-16) | 16 (14-19) | 15 (13-17) | 16 (13-18) | 14 (11-15) |
| Median age at last moment of follow-up (years) | 28 (24-34) | 45 (36-53) | 36 (30-43) | 31 (24-40) | 19 (17-22) | 29 (22-40) | 32 (23-46) | 30 (25-39) |
| Diagnostic delay (months) | 7 (2.87-12.25) | N.A. | 1 (0-2) | 0 (0-2) | 1.67 (1.29-2.00) | 0 (0-2) | N.A. | 2 (1-3) |
| Ethnicity | Other (Persian) 180/180 (100%) | N.A. | N.A. | Caucasian 116/116 (100%) | Caucasian 83/83 (100%) | Caucasian 129/129 (100%) | Caucasian 160/162 (98.8%) Other 2/162 (1.2%) | Latin-American 85/85 (100%) |
| History of febrile seizures | 12/180 (6.7%) | 3/38 (7.9%) | 10/239 (4.2%) | 7/116 (6.0%) | 4/83 (4.8%) | 8/145 (5.5%) | 3/165 (1.8%) | 6/85 (7.1%) |
| Ever experienced status epilepticus | 6/180 (3.3%) | 3/38 (7.9%) | 1/239 (0.4%) | 6/116 (5.2%) | N.A. | N.A. | 0/165 (0%) | 0/85 (0%) |
| Developmental delay | 1/180 (0.6%) | 0/38 (0%) | N.A. | 2/114 (1.8%) | 0/83 (0%) | 1/16 (6.3%) | 0/165 (0%) | 1/85 (1.2%) |
| Neurological comorbidities | 37/180 (20.6%) | 10/38 (26.3%) | 16/239 (6.7%) | 1/116 (0.9%) | 15/83 (18.1%) | 0/145 (0%) | 10/165 (6.1%) | 19/85 (22.3%) |
| Psychiatric comorbidities | 71/180 (39.4%) | 13/24 (54.2%) | 9/9 (100%) | 20/116 (17.2%) | 10/83 (12.0%) | 5/145 (3.4%) | 47/165 (28.5%) | 27/85 (31.8%) |
| Family history of epilepsy | 57/180 (31.7%) | 19/38 (50.0%) | 59/238 (24.8%) | 41/116 (35.3%) | 24/83 (28.9%) | 30/71 (42.3%) | 45/162 (27.8%) | 29/85 (34.1%) |
| Myoclonic seizures | 180/180 (100%) | 38/38 (100%) | 239/239 (100%) | 116/116 (100%) | 50/83 (60.2%) | 145/145 (100%) | 165/165 (100%) | 85/85 (100%) |
| Generalised tonic-clonic seizures | 169/180 (93.9%) | 36/38 (94.7%) | 236/239 (98.7%) | 101/116 (87.1%) | 59/83 (71.1%) | 129/145 (89.0%) | 155/165 (94.0%) | 81/85 (95.3%) |
| Absence seizures | 64/180 (35.6%) | 7/38 (18.4%) | 95/239 (39.7%) | 45/116 (38.8%) | 22/83 (26.5%) | 43/145 (29.7%) | 77/165 (46.7%) | 53/85 (62.4%) |
| Three seizure types | 60/180 (33.3%) | 6/38 (15.8%) | 93/239 (38.9%) | 39/116 (33.6%) | 9/93 (10.8%) | 44/145 (30.3%) | 77/165 (46.7%) | 52/85 (61.2%) |
| History of childhood absence epilepsy (CAE) progressing to JME | 6/180 (3.3%) | 1/38 (2.6%) | 14/239 (5.9%) | 16/116 (13.8%) | 7/83 (8.4%) | N.A. | 13/149 (8.7%) | 12/85 (14.1%) |
| Praxis-induced seizures | N.A. | 3/3 (100%) | N.A. | 1/116 (0.9%) | N.A. | N.A. | 1/157 (0.6%) | 0/85 (0%) |
| Epileptiform focality on EEG | 8/180 (4.4%) | 7/33 (21.2%) | N.A. | 14/115 (12.2%) | 0/83 (0%) | N.A. | 0/150 (0%) | 12/85 (14.1%) |
| Photoparoxysmal response | 16/180 (8.9%) | 5/32 (15.6%) | 39/239 (12.1%) | 57/116 (49.1%) | 18/68 (26.5%) | 6/145 (4.1%) | 22/151 (14.6%) | 26/85 (30.6%) |
| Motor seizures during sleep | N.A. | 2/2 (100%) | N.A. | 7/116 (6.0%) | 0/83 (0%) | N.A. | N.A. | 41/85 (48.2%) |
| Catamenial epilepsy | 2/2 (100%) | 3/3 (100%) | N.A. | 15/71 (21.1%) | N.A. | N.A. | 3/97 (3.1%) | 13/58 (22.4%) |
| ASM reduction | 9/180 (5%) | 12/38 (32%) | 0/239 (0%) | 18/116 (16%) | 0/83 (0%) | 0/165 (0%) | 38/126 (30%) | 12/85 (14%) |
| Median age at start of ASM reduction (years) | 23 (20-25) | 33 (28-38) | N.A. | 21 (17-24) | N.A. | N.A. | 22 (18-30) | 30 (27-36) |
| Median epilepsy duration before remission (years) | 4 (2-10) | 12 (7-22) | N.A. | 3 (1-3) | N.A. | N.A. | 8 (3-11) | 19 (11-23) |
| Median seizure-free interval before start of ASM reduction (years) | 2 (1-3) | 8 (6-11) | N.A. | 4 (3-5) | N.A. | N.A. | 2 (1-8) | 3 (2-3) |
| > 10 tonic-clonic seizures before remission | 1/9 (11.1%) | 1/11 (9.1%) | N.A. | 0/18 (0%) | N.A. | N.A. | 6/32 (18.8%) | 3/13 (23.1%) |
| EEG abnormality before reduction of ASM | 5/9 (55.6%) | 2/8 (25%) | N.A. | 6/17 (35.3%) | N.A. | N.A. | 25/32 (78.1%) | 1/13 (7.7%) |
| Median number of ASMs used at start of reduction | 1 (1-1) | 1 (1-1) | 1 (1-1) | 1 (1-1) | N.A. | N.A. | 1 (1-1) | 1 (1-1) |

| Study | Höfler[9] | Japaridze[10] | Jayalakshmi[11] | Karakis[12] | Kwan[13] | Özkara[14] | Pietrafusa[15] | von Podewils[16] |
|---|---|---|---|---|---|---|---|---|
| Publication year | 2014 | 2016 | 2014 | 2014 | 2019 | 2019 | 2021 | 2014 |
| Study design | Retrospective | Prospective | Retrospective | Retrospective | Retrospective | Retrospective | Retrospective | Retrospective |
| Country | Austria | Georgia | India | USA | Australia | Turkey | Italy | Germany |
| Subjects | 172 | 78 | 205 | 8 | 64 | 230 | 98 | 43 |
| Male sex | 63/172 (36.6%) | 34/78 (43.6%) | 91/205 (44.4%) | 4/8 (50.0%) | 34/64 (53.1%) | 94/230 (40.9%) | 29/98 (29.6%) | 16/43 (37.2%) |
| Drug-resistant JME | 62/169 (36.7%) | N.A. | 49/205 (23.9%) | 3/8 (37.5%) | 19/56 (33.9%) | 94/230 (40.9%) | 9/81 (11.1%) | 18/38 (5.3%) |
| Median age at first seizure (years) | 15 (12-18) | 15 (13-17) | 15 (12-17) | 16 (14-17) | 15 (14-17.3) | 15 (12-17) | 15 (12-16) | 14 (10-16) |
| Median age at last moment of follow-up (years) | 32 (24-41) | 22 (18-29) | 27 (23-36) | 23 (21-29) | 30 (23-38) | 27 (22-33) | 29 (22-35) | 29 (22-35) |
| Diagnostic delay (months) | N.A. | 1 (0-2) | 1 (0-2) | 0 (0-0.03) | 1 (1-1) | 1 (1-4) | 0 (0-0) | 1 (0-2) |
| Ethnicity | Caucasian 172/172 (100%) | Caucasian 78/78 (100%) | Asian 205/205 (100%) | N.A. | Caucasian 49/64 (76.6%) Asian 1/64 (1.6%) Other 14/64 (21.8%) | Caucasian 230/230 (100%) | Caucasian 98/98 (100%) | Caucasian 43/43 (100%) |
| History of febrile seizures | 0/172 (0%) | 13/77 (16.7%) | 39/205 (19.0%) | N.A. | 3/60 (5.0%) | 40/230 (17.4%) | 8/98 (8.2%) | 1/29 (3.4%) |
| Ever experienced status epilepticus | 4/172 (2.3%) | 0/77 (0%) | 21/205 (10.2%) | 0/8 (0%) | 6/64 (9.4%) | 10/230 (4.3%) | 0/98 (0%) | 1/38 (2.6%) |
| Developmental delay | 1/172 (0.6%) | 0/77 (0%) | 8/205 (3.9%) | 0/8 (0%) | 2/64 (3.1%) | 0/230 (0%) | 2/98 (2.0%) | 0/42 (0%) |
| Neurological comorbidities | 1/172 (0.6%) | 3/78 (3.8%) | 7/205 (3.4%) | 0/8 (0%) | 19/64 (29.7%) | 9/230 (3.9%) | 6/98 (6.1%) | 6/43 (14.0%) |
| Psychiatric comorbidities | 16/172 (9.3%) | 3/77 (3.9)% | 22/205 (10.7%) | 1/8 (12.5%) | 28/63 (44.4%) | 34/230 (14.8%) | 15/98 (15.3%) | 6/43 (14.0%) |
| Family history of epilepsy | 65/172 (37.8%) | 9/77 (11.7%) | 81/205 (39.5%) | 0/8 (0%) | 17/60 (28.3%) | 118/230 (51.3%) | 38/98 (38.8%) | 8/30 (26.7%) |
| Myoclonic seizures | 172/172 (100%) | 78/78 (100%) | 205/205 (100%) | 8/8 (100%) | 64/64 (100%) | 230/230 (100%) | 98/98 (100%) | 40/43 (93.0%) |
| Generalised tonic-clonic seizures | 167/172 (97.1%) | 59/78 (75.6%) | 199/205 (97.1%) | 8/8 (100%) | 62/64 (96.9%) | 219/230 (95.2%) | 82/98 (83.7%) | 40/43 (93.0%) |
| Absence seizures | 62/172 (36.0%) | 16/78 (20.5%) | 49/205 (23.9%) | 0/8 (0%) | 20/64 (31.3%) | 32/230 (13.9%) | 26/98 (26.5%) | 26/43 (60.5%) |
| Three seizure types | 55/172 (32.0%) | 11/78 (14.1%) | 46/205 (22.4%) | 0/8 (0%) | 22/64 (34.4%) | 63/230 (27.4%) | 21/98 (21.4%) | 20/43 (46.5%) |
| History of childhood absence epilepsy (CAE) progressing to JME | 30/172 (17.4%) | 1/77 (1.3%) | 23/205 (11.2%) | 0/8 (0%) | 5/64 (7.8%) | 9/230 (3.9%) | 10/98 (10.2%) | 4/37 (10.8%) |
| Praxis-induced seizures | 0/172 (0%) | 0/77 (0%) | 78/205 (38.0%) | N.A. | 0/63 (0%) | 9/230 (3.9%) | 0/98 (0%) | 0/28 (0%) |
| Epileptiform focality on EEG | 5/172 (2.9%) | 37/78 (47.4%) | 58/205 (28.3%) | 2/8 (25.0%) | 3/63 (4.8%) | 13/230 (5.7%) | 11/98 (11.2%) | 5/38 (13.2%) |
| Photoparoxysmal response | 20/172 (11.6%) | 34/77 (44.2%) | 103/205 (50.2%) | N.A. | 17/59 (28.8%) | 30/230 (13.0%) | 18/98 (18.4%) | 6/39 (15.4%) |
| Motor seizures during sleep | 0/172 (0%) | 14/77 (18.2%) | 116/205 (56.6%) | N.A. | 8/64 (12.5%) | 50/230 (21.7%) | 10/98 (10.2%) | 6/22 (27.3%) |
| Catamenial epilepsy | 0/109 (0%) | 6/43 (14.0%) | 41/108 (38.0%) | N.A. | 10/19 (52.6%) | 40/136 (29.4%) | 3/69 (4.3%) | N.A. |
| ASM reduction | 19/172 (11%) | 0/78 (0%) | 90/205 (44%) | 1/8 (13%) | 13/64 (20%) | 13/230 (6%) | 19/98 (19%) | 11/43 (26%) |
| Median age at start of ASM reduction (years) | N.A. | N.A. | 25 (21-31) | 13 (13-13) | 26 (23-33) | 32 (30-37) | 20 (16-26) | 18 (17-21) |
| Median epilepsy duration before remission (years) | N.A. | N.A. | 8 (4-11) | 2 (2-2) | 8 (3-17) | 12 (1-19) | 12 (10-15) | 3 (1-4) |
| Median seizure-free interval before start of ASM reduction (years) | N.A. | N.A. | 4 (3-5) | 2 (2-2) | 3 (2-6) | 2 (1-2) | 4 (3-7) | 1 (0-5) |
| > 10 tonic-clonic seizures before remission | N.A. | N.A. | 26/89 (29.2%) | N.A. | 6/12 (50%) | 0/13 (0%) | 0/10 (0%) | 0/9 (0%) |
| EEG abnormality before reduction of ASM | N.A. | N.A. | 11/89 (12.4%) | 1/1 (100%) | 4/7 (57.1%) | 2/3 (66.6%) | 0/1 (0%) | 1/4 (25%) |
| Median number of ASMs used at start of reduction | N.A. | N.A. | 1 (1-1) | 1 (1-1) | 2 (1-2) | 1 (1-1) | 1 (1-1) | 1 (1-1) |

| Study | Seneviratne[17] | Syvertsen[18] | Szaflarski[19] | Radhakrishnan[20] | Viloria Alebesque[21] | Viswanathan[22] | Vorderwülbecke[23] | Zhang[24] |
|---|---|---|---|---|---|---|---|---|
| Publication year | 2017 | 2014 | 2010 | 2003 | 2020 | 2021 | 2017 | 2020 |
| Study design | Retrospective | Prospective | Retrospective | Retrospective | Retrospective | Retrospective | Retrospective | Prospective |
| Country | Australia | Norway | USA | India | Spain | India | Germany | China |
| Subjects | 21 | 69 | 101 | 155 | 20 | 44 | 59 | 100 |
| Male sex | 4/21 (19.0%) | 23/69 (33.3%) | 36/101 (35.6%) | 68/155 (43.9%) | 11/20 (55.0%) | 22/44 (50%) | 30/59 (50.8%) | 44/100 (44.0%) |
| Drug-resistant JME | 11/21 (52.4%) | 23/51 (45.1%) | N.A. | 5/10 (50%) | 5/20 (25.0%) | 4/44 (9.1%) | 6/57 (10.5%) | 7/71 (9.9%) |
| Median age at first seizure (years) | 15 (12-17) | 14 (12-16) | 14 (11-16) | 13 (12-17) | 15 (12-17) | 14.5 (10-17) | 14 (12-16) | 13 (11-17) |
| Median age at last moment of follow-up (years) | 34 (26-42) | 26 (20-30) | 30 (20-30) | 29 (22-35) | 45 (38-48) | 28 (25-33) | 61 (49-72) | 21 (19-24) |
| Diagnostic delay (months) | 0 (0-1) | 0 (0-1) | N.A. | 1 (0-4) | 1 (0.25-4) | 3 (0-8) | N.A. | 2 (1-3) |
| Ethnicity | Caucasian 20/21 (95.2%) Asian 1/21 (4.8%) | Caucasian 65/69 (94.2%) Asian 4/69 (5.8%) | N.A. | Asian 155/155 (100%) | Caucasian 20/20 (100%) | Asian 44/44 (100%) | N.A. | Asian 100/100 (100%) |
| History of febrile seizures | 2/21 (9.5%) | 7/69 (10.1%) | N.A. | 30/155 (19.4%) | 0/20 (0%) | 4/44 (9.1%) | N.A. | 5/100 (5.0%) |
| Ever experienced status epilepticus | 3/21 (14.3%) | 0/69 (0%) | N.A. | 7/155 (4.5%) | 0/20 (0%) | 0/44 (0%) | N.A. | 0/100 (0%) |
| Developmental delay | 1/21 (4.8%) | 0/69 (0%) | N.A. | 1/154 (0.6%) | 0/20 (0%) | 0/44 (0%) | 0/26 (0%) | 0/100 (0%) |
| Neurological comorbidities | 0/21 (0%) | 0/69 (0%) | 0/101 (0%) | 14/155 (9.0%) | 3/20 (15%) | 1/44 (2.3%) | 7/59 (11.9%) | 6/100 (6.0%) |
| Psychiatric comorbidities | 6/21 (28.6%) | 37/69 (53.6%) | N.A. | 24/155 (15.5%) | 5/20 (25.0%) | 3/3 (100%) | 14/47 (29.8%) | N.A. |
| Family history of epilepsy | 12/21 (57.1%) | 23/69 (33.3%) | 52/99 (52.5%) | 43/155 (27.7%) | 9/20 (45%) | 9/39 (23.0%) | 5/59 (8.5%) | 3/3 (100%) |
| Myoclonic seizures | 21/21 (100%) | 69/69 (100%) | 100/101 (99.0%) | 155/155 (100%) | 20/20 (100%) | 44/44 (100%) | 59/59 (100%) | 100/100 (100%) |
| Generalised tonic-clonic seizures | 20/21 (95.2%) | 64/69 (92.8%) | 94/101 (93.1%) | 138/155 (89.0%) | 19/20 (95%) | 42/44 (95.5%) | 59/59 (100%) | 84/100 (84.0%) |
| Absence seizures | 6/21 (28.6%) | 23/69 (33.3%) | 48/101 (47.5%) | 31/155 (20.0%) | 4/20 (20.0%) | 4/44 (9.1%) | 13/59 (22.0%) | 22/100 (22.0%) |
| Three seizure types | 6/21 (28.6%) | 19/69 (27.5%) | 44/101 (43.6%) | 24/155 (15.5%) | 4/20 (20.0%) | 4/44 (9.1%) | 13/59 (22.0%) | 16/100 (16.0%) |
| History of childhood absence epilepsy (CAE) progressing to JME | 1/21 (4.8%) | 7/69 (10.1%) | 22/101 (21.8%) | 5/155 (3.2%) | 1/20 (5.0%) | N.A. | N.A. | 6/100 (6.0%) |
| Praxis-induced seizures | 0/7 (0%) | 4/69 (5.8%) | N.A. | 4/155 (2.6%) | N.A. | 0/44 (0%) | N.A. | 3/100 (3.0%) |
| Epileptiform focality on EEG | 6/21 (28.6%) | 10/69 (14.5%) | 27/101 (26.7%) | 80/155 (51.6%) | 4/20 (20.0%) | 3/44 (6.8%) | 7/59 (11.9%) | 13/33 (39.4%) |
| Photoparoxysmal response | 6/21 (28.6%) | 27/69 (39.1%) | N.A. | 14/155 (9.0%) | 5/20 (25.0%) | 0/44 (0%) | 16/59 (27.1%) | N.A. |
| Motor seizures during sleep | 2/21 (9.5%) | 2/69 (2.9%) | N.A. | 4/155 (2.6%) | 3/20 (15%) | 0/44 (0%) | N.A. | 1/100 (1.0%) |
| Catamenial epilepsy | N.A. | 4/46 (8.7%) | N.A. | 10/84 (11.9%) | 0/9 (0%) | 5/5 (100%) | N.A. | 1/56 (1.8%) |
| ASM reduction | 4/21 (19%) | 36/69 (52%) | 0/101 (0%) | 36/155 (23%) | 4/20 (20%) | 0/44 (0%) | 14/59 (24%) | 21/100 (21%) |
| Median age at start of ASM reduction (years) | 30 (27-40) | 19 (17-24) | N.A. | 22 (19-31) | 33 (26-38) | N.A. | 40 (34-46) | 22 (18-25) |
| Median epilepsy duration before remission (years) | 13 (8-24) | 3 (1-9) | N.A. | 10 (6-18) | 3 (3-6) | N.A. | 25.5 (18-32) | 5 (3-9) |
| Median seizure-free interval before start of ASM reduction (years) | 6 (5-9) | 1 (0-3) | N.A. | 4 (2-4) | 9 (6-13) | N.A. | 8 (4-12) | 3 (1-4) |
| > 10 tonic-clonic seizures before remission | 1/4 (25%) | 0/34 (0%) | N.A. | 3/13 (23.1%) | 0/4 (0%) | N.A. | N.A. | 4/18 (22.2%) |
| EEG abnormality before reduction of ASM | 2/3 (66.6%) | 2/8 (25%) | N.A. | 2/13 (15.4%) | 0/4 (0%) | N.A. | N.A. | 3/9 (33.3%) |
| Median number of ASMs used at start of reduction | 1 (1-1) | 1 (1-1) | N.A. | 2 (1-2) | 1 (1-1) | N.A. | N.A. | 1 (1-1) |

Supplementary Table 2: Overview of individuals and clinical variables from each of the included cohorts. Data are displayed as prevalence (%) or median (IQR) and split per study. Study names are the surname of the first author of the original study, or the last name when a different author provided IPD.

| First author and publication year | Study design | Number of people with JME | Drug resistance* | Pubmed ID |
|---|---|---|---|---|
| Camfield 2009[25] | Retrospective | 19 | 3/19 (15.8%) | 19786695 |
| Gomez-Ibañez 2017[26] | Retrospective | 69 | 35/69 (50.7%) | 28157601 |
| Guaranha 2011[27] | Retrospective | 65 | 40/65 (61.5%) | 21041102 |
| Martinez-Juárez 2006[28] | Prospective | 222 | 107/222 (48.2%) | 16520331 |
| Mehndiratta 2002[29] | Retrospective | 103 | 20/103 (19.4%) | 12237068 |
| Nicolson 2004[30] | Retrospective | 341 | 171/341 (50.1%) | 14707312 |
| Vollmar 2011[31] | Retrospective | 30 | 16/30 (53.3%) | 21616969 |
| Ashmawi 2017[32] | Retrospective | 69 | 20/69 (28.9%) | 28542735 |
| Sapio 2015[33] | Retrospective | 53 | 28/53 (52.8%) | 25875328 |
| Healy 2018[34] | Retrospective | 145 | 32/145 (22.1%) | 29807291 |
| Sánchez-Zapata 2019[35] | Retrospective | 145 | 51/145 (35.2%) | 31762000 |
| Gelisse 2001[36] | Retrospective | 140 | 24/140 (17.1%) | 11160477 |
| Landvogt 2010[37] | Retrospective | 12 | 5/12 (41.7%) | 20384765 |
| Asconapé 1984[38] | Retrospective | 11 | 5/11 (45.5) | 6420145 |
| Panayiotopoulos 1994[39] | Prospective | 50 | 16/50 (32.0%) | 8156946 |
| Chakravarty 2007[40] | Retrospective | 200 | 48/200 (24.0%) | 17218118 |
| Fernando-Dongas 2000[41] | Retrospective | 33 | 10/33 (30.3%) | 10985993 |
| Wu et al., 2018[42] | Retrospective | 124 | 57/124 (46.0%) | 29047147 |
| Choi 2020[43] | Retrospective | 199 | 48/199 (24.1%) | 32759205 |
| Chen 2020[44] | Retrospective | 63 | 23/69 (33.3%) | 32607028 |
| Takagi 2018[45] | Retrospective | 52 | NA | NA |
| Pedersen 1998[46] | Retrospective | 43 | 32/43 (74.4%) | 9531431 |
| Canevini 1992[47] | Prospective | 60 | 7/60 (11.7%) | 1344779 |
| Clement 1988[48] | Retrospective | 10 | 3/10 (30.0%) | 3140737 |
| Penry 1989[49] | Retrospective | 50 | 7/50 (14.0%) | 2506007 |
| Sharpe 1995[50] | Retrospective | 30 | 11/30 (36.7%) | 7854224 |
| Kleveland 1998[51] | Retrospective | 40 | 21/40 (52.6%) | 9548223 |
| Calleja 2001[52] | Retrospective | 22 | 4/22 (18.2%) | 11700996 |
| Siren 2002[53] | Retrospective | 13 | 8/13 (61.1%) | 12446224 |

Supplementary table 3: Details of eligible studies that were not included in the IPD meta-analysis. *The definition of drug resistance varied between studies.

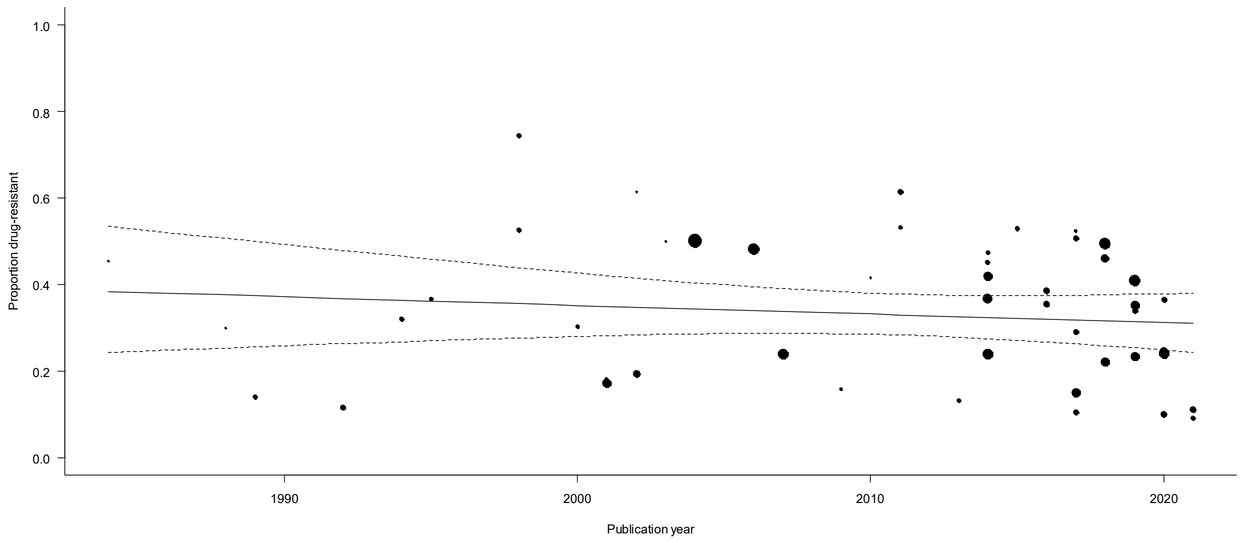


Supplementary figure 1: Meta-regression showing proportion of drug resistance by publication year. Each individual study is represented by a circle, whose size is proportional to the study sample size. This analysis also includes studies that were not included in our IPD meta-analysis.

| Study | Selection | | | | Comparability | | Outcome | | | Score |
|---|---|---|---|---|---|---|---|---|---|---|
| | Representativeness of the Exposed Cohort | Selection of the Non - Exposed Cohort | Ascertainment of Exposure | Outcome of Interest Was Not Present at Start of Study | Controls for the Most Important Factor (pseudo-refractory JME) | Controls for a Second Important Factor | Assessment of Outcome | Follow-Up Long Enough for Outcomes to Occur ( ≥5 years) | Adequacy of Follow Up of Cohorts | |
| Asadi-Pooya 2014 | * | * | | NA | | | | * | | 3 |
| Baykan 2008 | * | * | | NA | * | | | * | * | 5 |
| Cacao 2018 | * | * | | NA | | | | * | | 3 |
| Cerulli Irelli 2020 | * | * | * | NA | * | | | * | | 5 |
| Chowdhury 2016 | * | * | | NA | * | | | * | | 4 |
| EpiPGX, 2019 | * | * | * | NA | | | * | | | 4 |
| Gesche 2020 | * | * | | NA | * | | * | * | * | 6 |
| Özkara 2019 | * | * | * | NA | * | | | * | | 5 |
| Hernández-Venegas 2016 | * | * | | NA | | | | * | * | 4 |
| Höfler 2014 | * | * | | NA | | | | * | * | 4 |
| Japaridze 2016 | * | * | * | NA | | | | | | 3 |
| Jayalakshmi 2014 | * | * | * | NA | * | * | | * | * | 7 |
| Karakis 2014 | * | * | * | NA | * | | * | | | 5 |
| Pietrafusa et al., 2021 | * | * | * | NA | | | | * | * | 5 |
| Radhakrishnan 2003 | * | * | * | NA | | | | * | | 4 |
| Schneider-von Podewils 2014 | * | * | * | NA | | | | * | * | 5 |
| Seneviratne 2017 | * | * | * | NA | | | * | * | | 5 |
| Kwan 2019 | * | * | * | NA | | | * | | | 4 |
| Syvertsen 2014 | * | * | * | NA | | | | * | * | 5 |
| Szaflarski 2010 | * | * | * | NA | | | * | | * | 4 |
| Viloria-Alebesque, 2020 | * | * | | NA | | | | * | | 3 |
| Viswanathan 2021 | * | * | * | NA | | | | * | | 4 |
| Vorderwülbecke et al., 2017 | * | * | | NA | | | * | * | * | 5 |
| Zhang 2020 | * | * | * | NA | | | * | | * | 5 |

Supplementary Table 4: Risk of bias assessment using the Newcastle –Ottawa quality assessment scale for cohort studies. Studies can be attributed a maximum of one star (*) for each item. The total score is calculated as the sum of stars. A higher score indicates a better quality of the study.

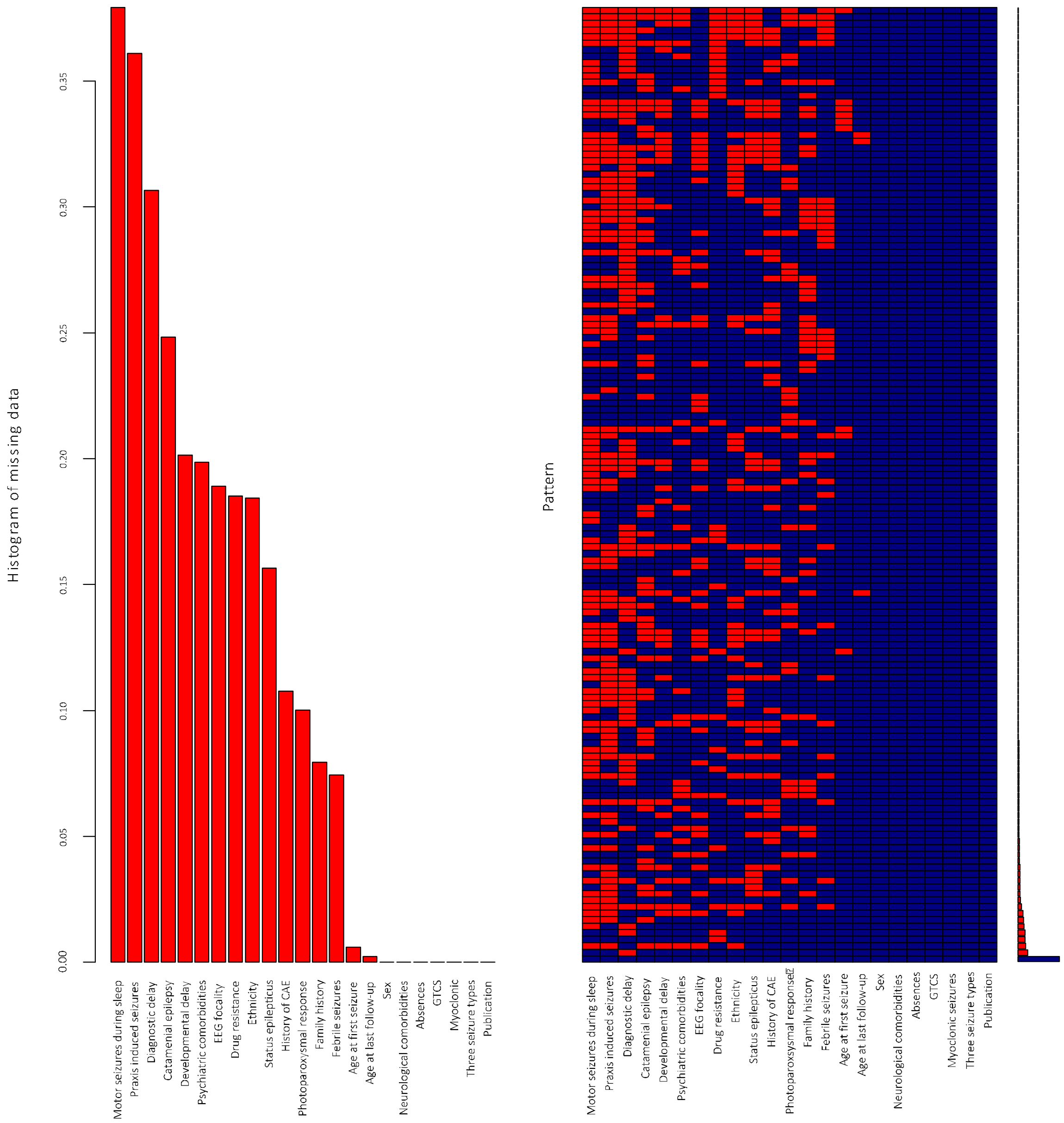


Supplementary Figure 2: Visualisation of missingness per variable before multiple imputation for all 2518 subjects included in analyses on drug resistance. The left plot displays the proportion of missingness per variable. The right plot shows patterns of missingness.

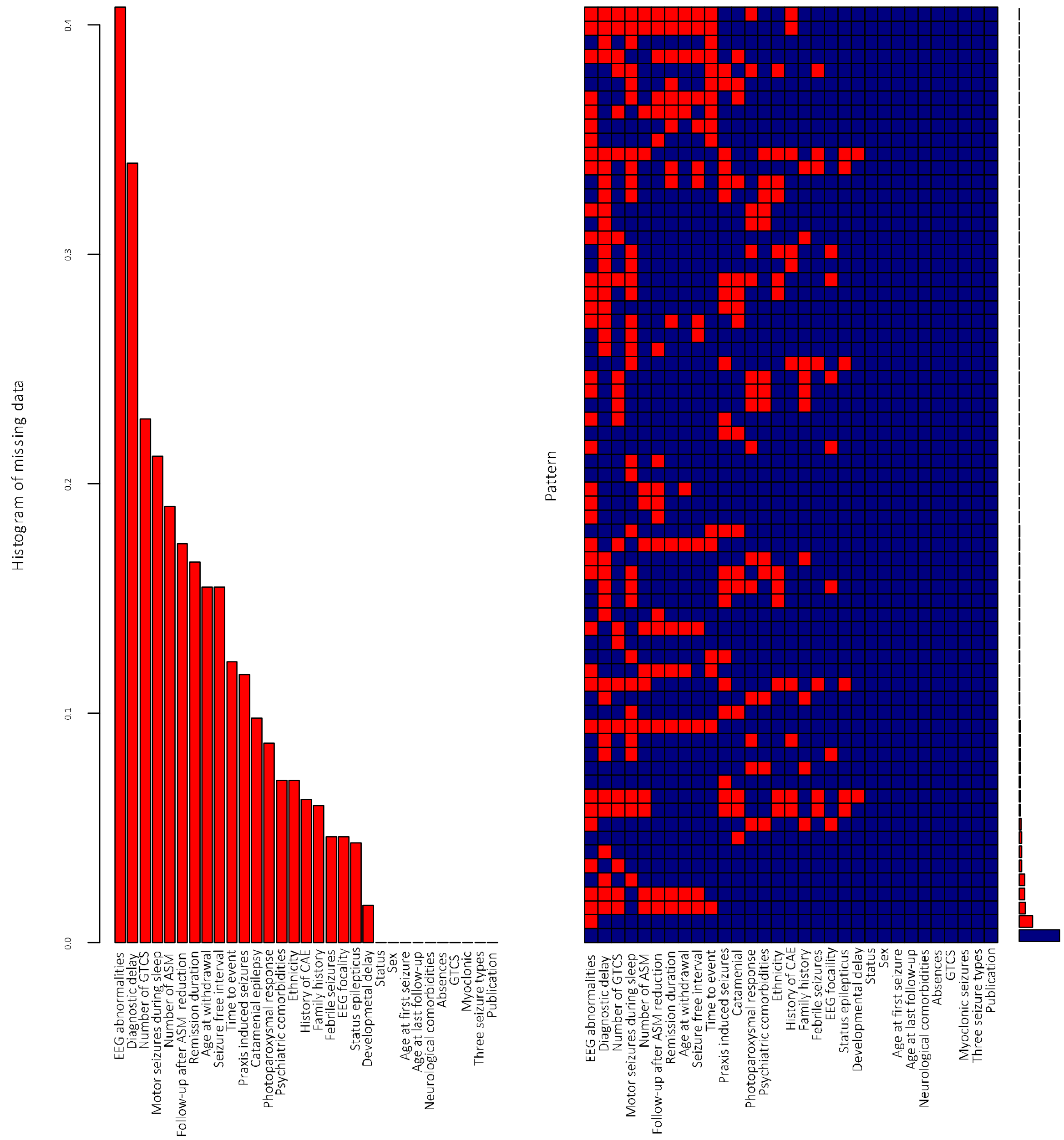


Supplementary Figure 3: Proportion and patterns of missingness per variable for 368 subjects included in analyses on seizure recurrence after ASM withdrawal.

| **Current ASM** | **n** | **percentage** |
|---|---|---|
| Multiple drugs | 805 | 34% |
| Valproic acid monotherapy | 826 | 35% |
| Levetiracetam monotherapy | 352 | 15% |
| Lamotrigine monotherapy | 154 | 7% |
| Topiramate monotherapy | 25 | 1% |
| Zonisamide monotherapy | 12 | 1% |
| Carbamazepine monotherapy | 11 | 0% |
| Phenytoine monotherapy | 5 | 0% |
| Clobazam monotherapy | 2 | 0% |
| Lacosamide monotherapy | 1 | 0% |
| Clonazepam monotherapy | 1 | 0% |
| Oxcarbazepine monotherapy | 1 | 0% |
| not taking any medication | 112 | 5% |

Supplementary Table 5: list of currently used medication. Percentage is based on the number of subjects (n=2356) of whom current ASM usage is known.

| **Past medication usage** | n | percentage |
|---|---|---|
| No previous ASM | 661 | 30% |
| One previous ASM | 727 | 33% |
| Two previous ASM | 412 | 19% |
| Three previous ASM | 232 | 10% |
| Four previous ASM | 108 | 5% |
| Five previous ASM | 30 | 1% |
| Six previous ASM | 30 | 1% |
| Seven previous ASM | 8 | 0% |
| Eight previous ASM | 5 | 0% |
| Nine previous ASM | 1 | 0% |
| Ten previous ASM | 1 | 0% |
| Eleven previous ASM | 1 | 0% |

Supplementary Table 6: list of previously used medication. Percentage is based on the number of subjects (n=2216) of whom previous ASM usage is known.

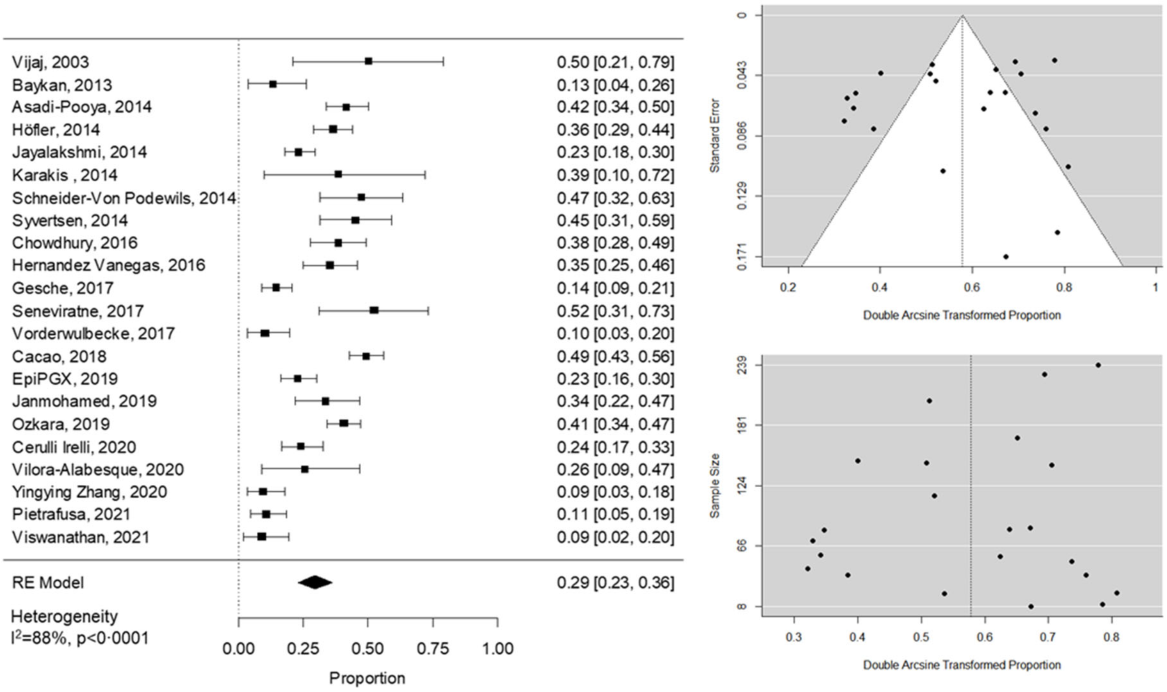


Supplementary figure 4: Meta-analysis of the proportion of drug-resistance, using a random-effects (RE) model. The proportion of drug-resistant people with JME (95% CI) is displayed. Funnel plots are displayed on the right.

| Predictor | Drug-responsive n (%) or median (IQR) | Drug-resistant n (%) or median (IQR) | No seizure recurrence n (%) or median (IQR) | Seizure recurrence n (%) or median (IQR) |
|---|---|---|---|---|
| Gender | | | | |
| Male | 563/1406 (40.0%) | 230/646 (35.6%) | 36/108 (33.3%) | 94/260 (36.2%) |
| Female | 843/1406 (60.0%) | 416/646 (64.4%) | 72/108 (66.7%) | 166/260 (63.8%) |
| Age at first seizure (years) | 15 (12-17) | 14 (12-16) | 15 (12-17) | 14 (11-16) |
| Age at last moment of follow-up (years) | 30 (23-39) | 30 (23-39) | 33 (24-44) | 30 (24-39) |
| Diagnostic delay (months) | 1 (0-3) | 1 (0-3) | 1 (0-3) | 1 (0-2) |
| Ethnicity | | | | |
| Caucasian | 775/1188 (65.2%) | 343/503 (68.2%) | 43/91 (47.3%) | 126/251 (50.2%) |
| Asian | 268/1188 (22.6%) | 66/503 (13.1%) | 45/91 (49.5%) | 106/251 (42.2) |
| Latin-American | 53/1188 (4.5%) | 29/503 (5.8%) | 1/91 (1.1%) | 11/251 (4.4%) |
| Other or admixed | 92/1188 (7.7%) | 65/503 (12.9%) | 2/91 (2.2%) | 8/251 (3.2%) |
| History of febrile seizures | | | | |
| Yes | 89/1341 (6.6%) | 65/629 (10.3%) | 9/96 (9.4%) | 25/255 (9.8%) |
| No | 1252/1341 (93.4%) | 564/629 (89.7%) | 87/96 (90.6%) | 230/255 (90.2%) |
| Ever experienced status epilepticus | | | | |
| Yes | 30/1692 (1.8%) | 32/571 (5.6%) | 1/96 (1%) | 10/256 (3.9%) |
| No | 1662/1692 (98.2%) | 539/571 (94.4%) | 95/96 (99%) | 246/256 (96.1%) |
| Developmental delay | | | | |
| Yes | 11/1158 (0.9%) | 8/490 (1.6%) | 1/104 (1.0%) | 3/258 (1.2%) |
| No | 1147/1158 (99.1%) | 482/490 (98.4%) | 103/104 (99.0%) | 255/258 (98.8%) |
| Neurological comorbidities | | | | |
| Yes | 211/1406 (15.0%) | 79/646 (12.2%) | 9/108 (8.3%) | 44/260 (16.9%) |
| No | 1195/1406 (85.0%) | 567/646 (87.8%) | 99/108 (91.7%) | 216/260 (83.1%) |
| Psychiatric comorbidities | | | | |
| Yes | 195/1166 (16.7%) | 156/518 (30.1%) | 15/88 (17.0%) | 61/254 (24.0%) |
| No | 971/1166 (83.3%) | 362/518 (69.9%) | 73/88 (83.0%) | 193/254 (76.0%) |
| Family history of epilepsy | | | | |
| Yes | 427/1276 (33.5%) | 229/609 (37.6%) | 39/92 (42.4%) | 86/254 (33.9%) |
| No | 849/1276 (66.5%) | 380/609 (62.4%) | 53/92 (57.6%) | 168/254 (66.1%) |
| Myoclonic seizures | | | | |
| Yes | 1384/1406 (98.4%) | 633/646 (98.0%) | 108/108 (100%) | 259/260 (100% |
| No | 22/1406 (1.6%) | 13/646 (2.0%) | 0/108 (0%) | 1/260 (0%) |
| Generalised tonic-clonic seizures (GTCS) | | | | |
| Yes | 1300/1406 (92.5%) | 615/646 (95.2%) | 100/108 (92.6%) | 245/260 (94.2%) |
| No | 106/1406 (7.5%) | 31/646 (4.8%) | 8/108 (7.4%) | 15/260 (5.8%) |
| Absence seizures | | | | |
| Yes | 341/1406 (24.3%) | 301/646 (46.6%) | 20/108 (18.5%) | 99/260 (38.1%) |
| No | 1065/1406 (75.7%) | 345/646 (53.4%) | 88/108 (81.5%) | 161/260 (61.9) |
| Three seizure types | | | | |
| Yes | 323/1406 (23.0%) | 308/646 (47.7%) | 17/108 (15.7%) | 86/260 (33.1%) |
| No | 1083/1406 (77.0%) | 338/646 (52.3%) | 91/108 (84.3%) | 174/260 (66.9%) |
| History of childhood absence epilepsy (CAE) progressing to JME | | | | |
| Yes | 77/1190 (6.5%) | 75/596 (12.6%) | 9/96 (9.4%) | 29/249 (11.6%) |
| No | 1113/1190 (93.5%) | 521/596 (87.4%) | 87/96 (90.6%) | 220/249 (88.4%) |
| Praxis-induced seizures | | | | |
| Yes | 61/927 (6.6%) | 36/358 (10.1%) | 7/86 (8.1%) | 28/239 (11.7%) |
| No | 866/927 (93.4%) | 322/358 (89.9%) | 79/86 (91.9%) | 211/239 (88.3%) |
| Epileptiform focality on EEG | | | | |
| Yes | 104/1121 (9.3%) | 75/481 (15.6%) | 14/104 (13.5%) | 43/247 (17.4%) |
| No | 1017/1121 (90.7%) | 406/481 (84.4%) | 90/104 (86.5%) | 204/247 (82.6%) |
| Photoparoxysmal response | | | | |
| Yes | 271/1310 (20.7%) | 144/622 (23.2%) | 25/93 (26.9%) | 77/243 (31.7%) |
| No | 1039/1310 (79.3%) | 478/622 (76.8%) | 68/93 (73.1%) | 166/243 (68.3%) |
| Motor seizures during sleep | | | | |
| Yes | 154/872 (17.7%) | 90/386 (23.3%) | 18/87 (20.7%) | 47/203 (23.2%) |
| No | 718/872 (82.3%) | 296/386 (76.7%) | 69/87 (79.3%) | 156/203 (76.8%) |
| Catamenial epilepsy* | | | | |
| Yes | 76/522 (14.6%) | 59/215 (27.4%) | 8/58 (13.8%) | 25/144 (17.4%) |
| No | 446/522 (85.4%) | 156/215 (72.6%) | 50/58 (86.2%) | 119/144 (82.6%) |
| Age at start of ASM reduction (years) | - | - | 29 (22-37) | 23 (18-30) |
| Epilepsy duration before remission (years) | - | - | 10 (4-16) | 8 (3-12) |
| Seizure-free interval before start of ASM reduction (years) | - | - | 6 (3-7) | 3 (2-5) |
| Number of GTCS before remission | | | | |
| < 10 | - | - | 66/76 (86.8%) | 168/208 (80.8%) |
| ≥ 10 | | | 10/76 (13.2%) | 40/208 (19.2%) |
| EEG abnormality before reduction of ASM | | | | |
| Yes | - | - | 11/55 (20.0%) | 56/163 (34.4%) |
| No | | | 44/55 (80.0%) | 107/163 (65.6%) |
| Number of ASMs used at start of reduction | - | - | 1 (1-1) | 1 (1-1) |

Supplementary table 7: Distributions of potential predictors in relation to drug resistance and seizure recurrence. The prevalence (%) is noted for categorical variables and the median (IQR) for numerical variables. Seizure recurrence is dichotomized, without taking time into account such as in a Cox proportional hazards survival model. Missing data differs per variable and proportions are calculated based on non-missing data. Catamenial epilepsy is a female-specific risk factor. Thus, we have calculated the proportion as a fraction of female subjects.

| | Sex | Age at first seizure | Age at last follow-up | Diagnostic delay | Ethnicity | History of febrile seizures | Status epilepticus | Developmental delay | Psychiatric comorbidities | Neurological comorbidities | Family history | Absences | GTCS | Myoclonic | Three seizure types | History of CAE | Praxis induced seizures | Catamenial epilepsy | EEG focality | Photoparoxysmal response | Motor seizures in sleep |
|---|---|---|---|---|---|---|---|---|---|---|---|---|---|---|---|---|---|---|---|---|---|
| **Sex** | 1.00 | 0.05 | 0.01 | 0.00 | 0.02 | 0.03 | -0.03 | 0.00 | -0.06 | 0.02 | -0.02 | -0.05 | 0.03 | -0.03 | -0.03 | -0.07 | 0.03 | -0.32 | 0.02 | -0.05 | 0.01 |
| **Age at first seizure** | 0.05 | 1.00 | 0.16 | -0.03 | -0.06 | -0.07 | -0.01 | 0.00 | 0.00 | 0.09 | -0.02 | -0.24 | 0.01 | -0.06 | -0.20 | -0.28 | -0.01 | -0.01 | -0.05 | -0.07 | 0.03 |
| **Age at last follow-up** | 0.01 | 0.16 | 1.00 | 0.20 | -0.04 | -0.05 | 0.06 | 0.04 | 0.11 | 0.10 | -0.01 | 0.06 | 0.14 | 0.10 | 0.09 | 0.03 | -0.01 | 0.01 | -0.01 | -0.03 | 0.07 |
| **Diagnostic delay** | 0.00 | -0.03 | 0.20 | 1.00 | 0.06 | 0.00 | 0.04 | 0.00 | 0.13 | 0.06 | -0.01 | 0.03 | 0.03 | 0.02 | 0.03 | 0.01 | -0.01 | 0.09 | -0.03 | -0.06 | 0.06 |
| **Ethnicity** | 0.02 | -0.06 | -0.04 | 0.06 | 1.00 | 0.06 | 0.06 | 0.04 | 0.02 | -0.06 | -0.05 | 0.01 | 0.05 | 0.09 | 0.00 | -0.01 | 0.20 | 0.09 | 0.23 | 0.07 | 0.21 |
| **History of febrile seizures** | 0.03 | -0.07 | -0.05 | 0.00 | 0.06 | 1.00 | 0.09 | 0.09 | 0.00 | -0.04 | 0.03 | -0.01 | 0.01 | 0.00 | 0.01 | 0.05 | 0.04 | -0.02 | 0.03 | 0.00 | 0.02 |
| **Status epilepticus** | -0.03 | -0.01 | 0.06 | 0.04 | 0.06 | 0.09 | 1.00 | 0.13 | 0.07 | -0.03 | 0.06 | 0.01 | 0.05 | 0.01 | 0.03 | 0.06 | 0.09 | -0.01 | 0.04 | 0.04 | 0.07 |
| **Developmental delay** | 0.00 | 0.00 | 0.04 | 0.00 | 0.04 | 0.09 | 0.13 | 1.00 | 0.04 | 0.00 | 0.04 | 0.02 | 0.03 | 0.01 | 0.03 | 0.10 | 0.14 | -0.01 | 0.11 | 0.04 | 0.04 |
| **Psychiatric comorbidities** | -0.06 | 0.00 | 0.11 | 0.13 | 0.02 | 0.00 | 0.07 | 0.04 | 1.00 | 0.15 | -0.01 | 0.13 | 0.05 | 0.05 | 0.14 | 0.03 | 0.02 | 0.06 | -0.02 | 0.00 | 0.03 |
| **Neurological comorbidities** | 0.02 | 0.09 | 0.10 | 0.06 | -0.06 | -0.04 | -0.03 | 0.00 | 0.15 | 1.00 | -0.02 | 0.08 | 0.01 | 0.01 | 0.10 | 0.02 | -0.07 | -0.07 | -0.08 | -0.06 | 0.03 |
| **Family history** | -0.02 | -0.02 | -0.01 | -0.01 | -0.05 | 0.03 | 0.06 | 0.04 | -0.01 | -0.02 | 1.00 | 0.02 | 0.00 | 0.00 | 0.02 | 0.05 | 0.07 | -0.02 | -0.02 | 0.01 | 0.00 |
| **Absences** | -0.05 | -0.24 | 0.06 | 0.03 | 0.01 | -0.01 | 0.01 | 0.02 | 0.13 | 0.08 | 0.02 | 1.00 | 0.00 | -0.01 | 0.88 | 0.36 | 0.03 | 0.00 | 0.01 | -0.01 | 0.05 |
| **GTCS** | 0.03 | 0.01 | 0.14 | 0.03 | 0.05 | 0.01 | 0.05 | 0.03 | 0.05 | 0.01 | 0.00 | 0.00 | 1.00 | 0.05 | 0.18 | -0.04 | 0.04 | 0.05 | 0.00 | 0.01 | 0.09 |
| **Myoclonic** | -0.03 | -0.06 | 0.10 | 0.02 | 0.09 | 0.00 | 0.01 | 0.01 | 0.05 | 0.01 | 0.00 | -0.01 | 0.05 | 1.00 | 0.08 | -0.02 | 0.03 | 0.03 | 0.05 | 0.01 | 0.04 |
| **Three seizure types** | -0.03 | -0.20 | 0.09 | 0.03 | 0.00 | 0.01 | 0.03 | 0.03 | 0.14 | 0.10 | 0.02 | 0.88 | 0.18 | 0.08 | 1.00 | 0.29 | 0.04 | 0.03 | 0.00 | 0.00 | 0.07 |
| **History of CAE** | -0.07 | -0.28 | 0.03 | 0.01 | -0.01 | 0.05 | 0.06 | 0.10 | 0.03 | 0.02 | 0.05 | 0.36 | -0.04 | -0.02 | 0.29 | 1.00 | 0.04 | -0.01 | -0.01 | 0.05 | 0.02 |
| **Praxis induced seizures** | 0.03 | -0.01 | -0.01 | -0.01 | 0.20 | 0.04 | 0.09 | 0.14 | 0.02 | -0.07 | 0.07 | 0.03 | 0.04 | 0.03 | 0.04 | 0.04 | 1.00 | 0.05 | 0.18 | 0.21 | 0.23 |
| **Catamenial epilepsy** | -0.32 | -0.01 | 0.01 | 0.09 | 0.09 | -0.02 | -0.01 | -0.01 | 0.06 | -0.07 | -0.02 | 0.00 | 0.05 | 0.03 | 0.03 | -0.01 | 0.05 | 1.00 | 0.01 | 0.02 | 0.11 |
| **EEG focality** | 0.02 | -0.05 | -0.01 | -0.03 | 0.23 | 0.03 | 0.04 | 0.11 | -0.02 | -0.08 | -0.02 | 0.01 | 0.00 | 0.05 | 0.00 | -0.01 | 0.18 | 0.01 | 1.00 | 0.05 | 0.07 |
| **Photoparoxysmal response** | -0.05 | -0.07 | -0.03 | -0.06 | 0.07 | 0.00 | 0.04 | 0.04 | 0.00 | -0.06 | 0.01 | -0.01 | 0.01 | 0.01 | 0.00 | 0.05 | 0.21 | 0.02 | 0.05 | 1.00 | 0.12 |
| **Motor seizures in sleep** | 0.01 | 0.03 | 0.07 | 0.06 | 0.21 | 0.02 | 0.07 | 0.04 | 0.03 | 0.03 | 0.00 | 0.05 | 0.09 | 0.04 | 0.07 | 0.02 | 0.23 | 0.11 | 0.07 | 0.12 | 1.00 |

Supplementary Table 8: Correlation table of potential predictors of drug resistance. Pearson correlation coefficients are displayed.

| Predictor | Full model | | Reduced model | |
|---|---|---|---|---|
| | OR (95%CI) | p-value | OR (95%CI) | p-value |
| History of febrile seizures | 1.65 (1.11-2.47) | 0.015 | 1.62 (1.09-2.41) | 0.018 |
| Status epilepticus | 1.63 (0.88-3.03) | 0.12 | 1.71 (0.94-3.13) | 0.079 |
| Psychiatric comorbidities | 1.94 (1.49-2.54) | <0.0001 | 1.96 (1.51-2.56) | <0.0001 |
| Family history of epilepsy | 1.23 (0.97-1.54) | 0.08 | 1.23 (0.98-1.54) | 0.07 |
| Three seizure types | 2.34 (1.47-3.74) | 0.00038 | 2.84 (2.23-3.60) | <0.0001 |
| Catamenial epilepsy | 1.97 (1.30-2.98) | 0.0020 | 2.02 (1.37-2.97) | <0.0001 |
| EEG focality | 2.31 (1.58-3.40) | <0.0001 | 2.40 (1.64-3.50) | <0.0001 |
| History of childhood absence epilepsy | 1.30 (0.83-2.00) | 0.24 | 1.41 (0.92-2.18) | 0.12 |
| Asian ethnicity* | 0.34 (0.18-0.64) | 0.0013 | 0.38 (0.21-0.72) | 0.0039 |
| Latin-American ethnicity* | 0.60 (0.23-1.57) | 0.30 | 0.73 (0.28-1.90) | 0.52 |
| Other ethnicity* | 1.04 (0.47-2.35) | 0.91 | 1.12 (0.51-2.45) | 0.77 |
| Sex | 1.02 (0.82-1.29) | 0.83 | - | - |
| Age at first seizure | 0.99 (0.97-1.01) | 0.35 | - | - |
| Developmental delay | 0.87 (0.30-2.54) | 0.80 | - | - |
| Absences | 1.21 (0.75-1.95) | 0.44 | - | - |
| GTCS | 1.03 (0.61-1.71) | 0.91 | - | - |
| Praxis induced seizures | 1.31 (0.76-2.27) | 0.34 | - | - |
| Photoparoxysmal response | 1.19 (0.90-1.57) | 0.21 | - | - |
| Motor seizures during sleep | 1.49 (1.08-2.06) | 0.014 | - | - |
| Diagnostic delay | 1.10 (0.92-1.31) | 0.30 | - | - |

Supplementary Table 9: Multivariable predictors of drug resistance. The full model includes all 18 variables with p<0.2 in univariable analyses. The reduced model includes the nine variables with independent predictive value, after backward selection of the least contributing variables. OR: odds ratio. *Caucasian ethnicity was set as reference.

| | Full data with multiple imputations | | Data restricted to complete cases | |
|---|---|---|---|---|
| **Predictor** | **OR (95%CI); univariable model (n=2518)** | **OR (95%CI); multivariable model (n=2518)** | **OR (95%CI); univariable model†** | **OR (95%CI); multivariable model (1163)** |
| History of febrile seizures | 1.57 (1.14 - 2.17) | 1.62 (1.09-2.41) | 1.73 (1.21 - 2.47) | 1.72 (1.10 - 2.69) |
| Status epilepticus | 2.29 (1.37 - 3.84) | 1.71 (0.94-3.13) | 2.58 (1.51 – 4.41) | 1.21 (0.61 - 2.41) |
| Psychiatric comorbidities | 2.27 (1.78 - 2.89) | 1.96 (1.51-2.56) | 2.29 (1.76 – 2.99) | 2.51 (1.78 - 3.56) |
| Family history of epilepsy | 1.22 (0.99 - 1.51) | 1.23 (0.98-1.54) | 1.22 (0.99 – 1.51) | 1.08 (0.82 - 1.44) |
| Three seizure types | 3.26 (2.64 - 4.02) | 2.84 (2.23-3.60) | 3.24 (2.62 – 4.01) | 2.84 (2.10 - 3.85) |
| Catamenial epilepsy | 2.13 (1.44 - 3.16) | 2.02 (1.37-2.97) | 2.18 (1.48 – 3.21) | 1.82 (1.18 - 2.80) |
| EEG focality | 2.23 (1.59 - 3.13) | 2.40 (1.64-3.50) | 2.34 (1.64 – 3.35) | 3.05 (1.97 - 4.71) |
| History of childhood absence epilepsy | 2.34 (1.67 - 3.29) | 1.41 (0.92-2.18) | 2.45 (1.72 – 3.48) | 1.33 (0.85 - 2.08) |
| Asian ethnicity* | 0.46 (0.24 - 0.88) | 0.38 (0.21-0.72) | 0.49 (0.24 – 0.99) | 0.84 (0.31 - 2.30) |
| Latin-American ethnicity* | 1.31 (0.52 - 3.31) | 0.73 (0.28-1.90) | 1.23 (0.37 – 4.01) | 0.86 (0.24 - 3.04) |
| Other ethnicity* | 1.01 (0.32 - 3.20) | 1.12 (0.51-2.45) | 1.47 (0.63 – 3.40) | 1.33 (0.48 - 3.66) |

Supplementary table 10: Comparison of univariable and multivariable associations of predictors in the final model with drug resistance using the full multiply imputed dataset and model restricted to complete cases. OR: odds ratio. *Caucasian ethnicity was set as reference. † Missingness differs per variable.

| **Study** | **AUC** |
|---|---|
| Asadi-Pooya 2014 | 0.61 |
| Baykan 2008 | 0.78 |
| Cacao 2018 | 0.74 |
| Cerulli Irelli 2020 | 0.84 |
| Chowdhury 2016 | 0.56 |
| EpiPGX, 2019 | 0.70 |
| Gesche 2020 | 0.75 |
| Özkara 2019 | 0.67 |
| Hernández-Vanegas 2016 | 0.62 |
| Höfler 2014 | 0.64 |
| Japaridze 2016 | 0.67 |
| Jayalakshmi 2014 | 0.69 |
| Karakis 2014 | 0.69 |
| Pietrafusa et al., 2021 | 0.76 |
| Radhakrishnan 2003 | 0.71 |
| Schneider-von Podewils 2014 | 0.67 |
| Seneviratne 2017 | 0.62 |
| Kwan 2019 | 0.83 |
| Syvertsen 2014 | 0.67 |
| Szaflarski 2010 | 0.76 |
| Viloria-Alebesque, 2020 | 0.76 |
| Viswanathan 2021 | 0.73 |
| Vorderwülbecke et al., 2017 | 0.58 |
| Zhang 2020 | 0.76 |

Supplementary table 11: Predictive performance (AUC) for each individual left-out cohort. The AUC was calculated by training the statistical model on all datasets except the left out cohort, after which predictions were calculated on the left-out cohort.

| | Drug-resistance | Freedom of any seizure the last year | Freedom of any seizure the last 2 years | Freedom of GTCS seizure the last year | Freedom of GTCS seizure the last 2 years | Freedom of GTCS seizure the last 5 years |
|---|---|---|---|---|---|---|
| **Drug-resistance** | 1.00 | -0.90 | -0.72 | -0.71 | -0.60 | -0.37 |
| **Freedom of any seizure the last year** | -0.90 | 1.00 | 0.75 | 0.75 | 0.56 | 0.34 |
| **Freedom of any seizure the last 2 years** | -0.72 | 0.75 | 1.00 | 0.56 | 0.79 | 0.48 |
| **Freedom of GTCS seizure the last year** | -0.71 | 0.75 | 0.56 | 1.00 | 0.71 | 0.42 |
| **Freedom of GTCS seizure the last 2 years** | -0.60 | 0.56 | 0.79 | 0.71 | 1.00 | 0.59 |
| **Freedom of GTCS seizure the last 5 years** | -0.37 | 0.34 | 0.48 | 0.42 | 0.59 | 1.00 |

Supplementary Table 12: Correlation table of different outcome measures. Pearson correlation coefficients are displayed.

| | Attempted withdrawal (n=368) | Not attempted withdrawal (n=2150) | P-value |
|---|---|---|---|
| Age at last follow-up | 31.0 (24.0-39.25) | 29.0 (22.3-38.0) | 0.025 |
| Female sex | 238/368 (64.7%) | 1302/2150 (60.5%) | 0.12 |
| History of febrile seizures | 34/351 (9.7%) | 171/1980 (8.6%) | 0.51 |
| Status epilepticus | 11/352 (3.1%) | 57/1772 (3.2%) | 0.94 |
| Psychiatric comorbidities | 76/342 (22.2%) | 340/1676 (20.2%) | 0.42 |
| Family history of epilepsy | 125/346 (36.1%) | 671/1972 (34.0%) | 0.49 |
| Three seizure types | 103/368 (28.0%) | 645/2150 (30.0%) | 0.46 |
| Catamenial epilepsy | 33/202 (16.3%) | 123/713 (17.2%) | 0.76 |
| EEG focality | 57/351 (16.2%) | 268/1691 (15.8%) | 0.84 |
| History of childhood absence epilepsy | 38/345 (11.1%) | 155/1902 (8.1%) | 0.08 |
| Caucasian ethnicity | 169/342 (49.4%) | 1094/1712 (64%) | <0.0001 |
| Asian ethnicity | 151/342 (44.2%) | 359/1712 (21.0%) | <0.0001 |
| Latin-American ethnicity | 12/342 (3.5%) | 73/1712 (4.3%) | 0.53 |
| Other ethnicity | 10/342 (3.4%) | 186/1712 (10.8%) | <0.0001 |

Supplementary table 13: Comparison between the subset of people who attempted to withdraw ASM treatment and those who had not attempted this.

| | Full model | | Reduced model | |
|---|---|---|---|---|
| **Predictor** | **HR (95%CI)** | **p-value** | **HR (95%CI)** | **p-value** |
| Age at start of ASM reduction | 0.98 (0.95-1.00) | 0.066 | 0.97 (0.95 - 0.98) | <0.0001 |
| Seizure free interval before withdrawal* | 0.69 (0.52-0.91) | 0.010 | 0.69 (0.53 - 0.91) | 0.0083 |
| Number of ASM used before start of ASM reduction | 1.39 (1.02-1.88) | 0.038 | 1.44 (1.07– 1.95) | 0.018 |
| Psychiatric comorbidities | 1.23 (0.89-1.70) | 0.21 | - | - |
| Epilepsy duration before remission | 0.99 (0.96-1.01) | 0.35 | - | - |
| History of febrile seizures | 0.68 (0.42-1.10) | 0.12 | - | - |
| Photoparoxysmal response | 1.31 (0.95-1.80) | 0.10 | - | - |
| Absence seizures | 1.37 (0.74-2.57) | 0.32 | - | - |
| Three seizure types | 0.95 (0.51-1.77) | 0.87 | - | - |

Supplementary Table 14: Multivariable predictors of recurrence of any seizure after ASM withdrawal. The full model includes all variables with p<0.2 in univariable analyses. The reduced model includes the three variables with independent predictive value, after backward selection of the least contributing variables. HR: hazard ratio. * Cube root transformed.

| | Full data with multiple imputations | | Data restricted to complete cases | |
|---|---|---|---|---|
| **Predictor** | **HR (95%CI); univariable model (n=368)** | **HR (95%CI); multivariable model (n=368)** | **HR (95%CI); univariable model †** | **HR (95%CI); multivariable model (n=282)** |
| Age at start of ASM reduction | 0.96 (0.95 - 0.98) | 0.97 (0.95 - 0.98) | 0.96 (0.95 - 0.98) | 0.97 (0.96 - 0.99) |
| Seizure free interval before withdrawal* | 0.58 (0.46 - 0.74) | 0.69 (0.53 - 0.91) | 0.55 (0.43 – 0.70) | 0.67 (0.50 - 0.91) |
| Number of ASM used before start of ASM reduction | 1.36 (1.01 - 1.82) | 1.44 (1.07– 1.95) | 1.36 (1.02 – 1.80) | 1.41 (1.04 - 1.90) |

Supplementary table 15: Comparison of multivariable prediction model of drug resistance using the multiply imputed dataset (n=368) and model restricted to complete cases (n=282). HR: hazard ratio. * Cube root transformed. † Missingness differs per variable.

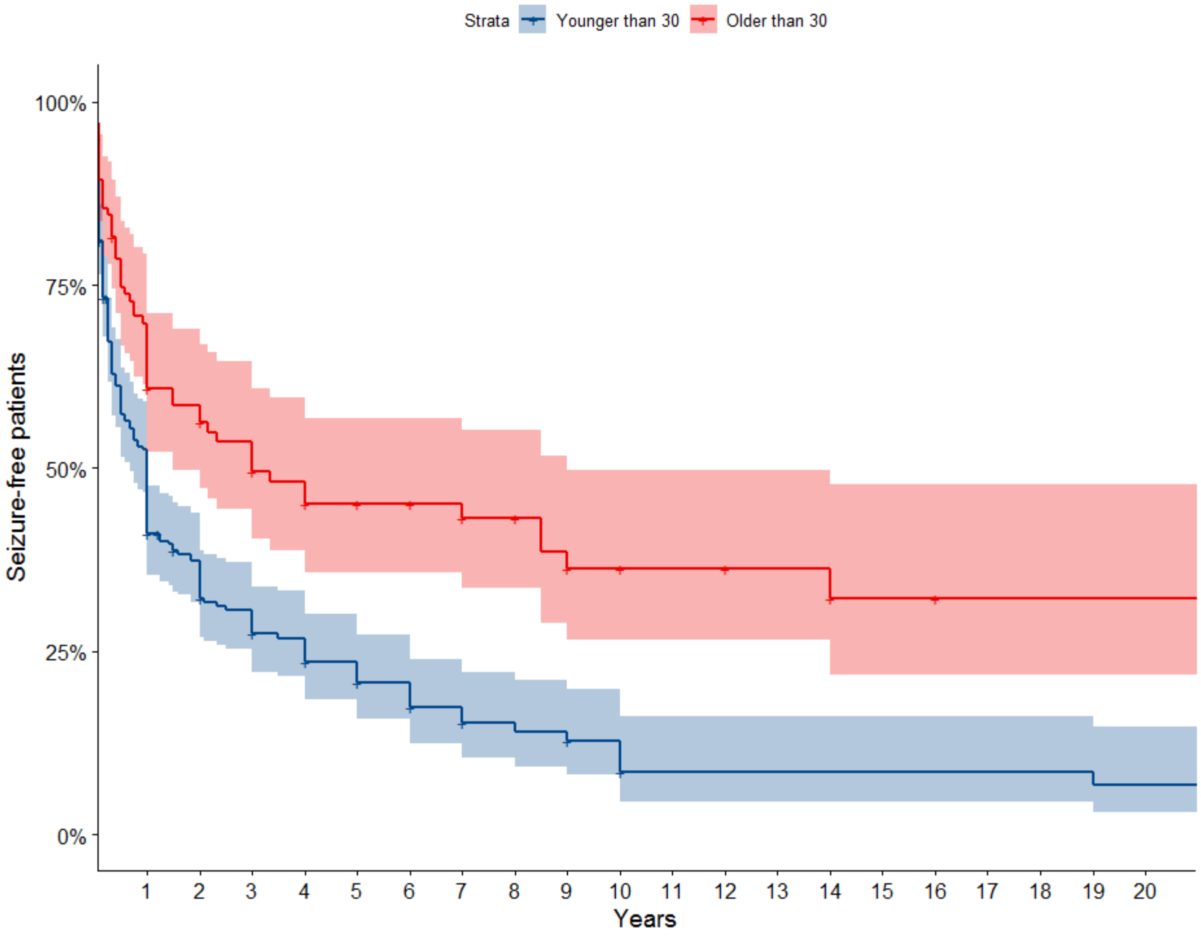


Supplementary Figure 5: Recurrence of any seizure after ASM withdrawal, split by age at withdrawal older or younger than 30 years. The 95% confidence interval is displayed in shaded colours.

| | **Full model** | | **Reduced model** | |
|---|---|---|---|---|
| **Predictor** | **HR (95%CI)** | **p-value** | **HR (95%CI)** | **p-value** |
| Age at withdrawal | 0.98 (0.96 - 1.00) | 0.035 | 0.98 (0.96 - 1.00) | 0.034 |
| Seizure free interval before withdrawal* | 0.81 (0.59-1.10) | 0.18 | 0.87 (0.65 - 1.18) | 0.38 |
| Number of ASM used at start of ASM reduction | 1.32 (0.96-1.81) | 0.093 | 1.56 (1.09 – 2.24) | 0.016 |
| More than 10 GTCS before withdrawal | 1.58 (1.09-2.30) | 0.018 | 1.34 (0.95 - 1.88) | 0.096 |
| Catamenial epilepsy | 1.74 (1.09-2.78) | 0.022 | - | - |

Supplementary Table 16: Multivariable predictors of recurrence of GTCS after ASM withdrawal. The full model includes all variables with p<0.2 in univariable analyses. The reduced model includes the four variables with independent predictive value, after backward selection of the least contributing variables. HR: hazard ratio. * Cube root transformed.

## Supplementary methods

**Data transformations:**

We sent a standardized data entry sheet for individual patient data to all collaborators, including explanations of each variable (**Supplementary Table 1**). Two authors (RS and D-AT) manually checked all data entries for inconsistencies, and potential discrepancies were resolved after discussion.

Skewness of numerical variables was assessed with the 'moments' R package. Approximate normality was achieved by cube root transformation of the variables 'diagnostic delay' and 'seizure-free interval before reduction of ASM treatment', and log transformation of 'age at last follow-up'.

For categorical variables with >2 categories, we attempted to reduce the number of categories as much as possible without losing information. For the variable ethnicity, we merged subjects of African (n=3) and admixed ancestry (n=180) with the category "other" since they constituted few people and merging gave a better fit (i.e., lower Akaike Information criterion [AIC]). Catamenial epilepsy is a female-specific risk factor. We attempted two ways to model this; by creating a variable with three categories ("yes", "no" and "male") and by creating a binary variable ("yes" or "no") where each male was converted to "no". When assessing this variable in multivariable models of drug resistance, the binary variant gave a better fit (i.e., a lower AIC), after which this option was chosen for further analyses.

**Statistical methods**

The missing data was imputed using Multiple Imputations with Chained Equations (MICE),[54] which decreases bias and improves statistical estimates compared to deleting missing cases.[55–57] We imputed predictors and outcome variables, which is the preferred method and does not induce bias.[58,59] We performed twenty imputations, using the default settings of MICE: numerical variables were imputed with predictive mean matching and categorical variables were imputed with logistic regression. Baseline characteristics were computed using non-imputed data. Random-effects meta-analysis was performed on non-imputed summary data to assess the proportion of drug-resistant JME. All other analyses in this manuscript are based on twenty imputations, which were subsequently pooled.

Drug resistance

Prediction models for drug-resistance and seizure-freedom in the last years of follow-up were computed with mixed-effects logistic regression, using the generalized linear mixed-effects model (glmer). All analyses were corrected for between-cohort heterogeneity by including the cohort as a random-effects variable. Univariate analyses were computed on the imputed dataset, after which all predictors with p<0.20 were selected for creation of a multivariable model. The multivariable model was further reduced removing the least contributing predictors as assessed with the AIC, until an optimal model with the least number of predictors was found. We created a nomogram based on the reduced multivariable model.

Furthermore, we plotted the observed vs predicted probabilities to assess calibration. We performed internal-external cross-validation (IECV) by iteratively leaving one cohort out of the analyses and predicting the chance of drug resistance on the left-out cohort, based on all remaining data. In this way, we calculated probabilities of drug resistance for each subject in the dataset, without any of the predictions being based on the cohort in which they were recruited. Next, we calculated the area under the receiver operator curve (AUC) based on the merged probabilities for all subjects from the IECV. As sensitivity analyses, we performed the same IECV analyses to predict seizure freedom in the last one, two and five years, and specifically assessing freedom of GTCS in the last one, two and five years. We performed analyses on seizure freedom in the last two and five years only for subjects with at least two years (n=1966) or five years (n=1565) follow-up respectively. Analyses on freedom of GTCS were limited to subjects who had ever experienced GTCS (n=2322).

Seizure-recurrence after ASM withdrawal

A subset of 368 subjects who attempted to withdraw ASM were selected for analyses on recurrence of seizures. Cox-proportional hazards models were used to assess predictors of seizure recurrence and create the prediction model for recurrence at two and five years after withdrawal. We compared a fixed-effects and mixed-effects random effects Cox-proportional hazards model, and found that adding cohort as a random-effects variable did not improve the model fit (as assessed with the AIC), after which we chose to perform all analyses using fixed-

effects models. The assumption of linearity for continuous variables was assessed by plotting Martingale residuals. The assumption of proportional hazards was assessed by a statistical test and manual inspection of a plot with time vs beta-coefficient.[60] Predictors with a univariate p<0.2 were used to create a multivariable prediction model, after which backwards selection based on the AIC was used to remove the least contributing variables. We excluded age at last follow-up as a potential predictor in the multivariable model, since this variable is not known prior to withdrawal and is highly collinear with age at withdrawal ($R^2$=0.8). The final multivariable model was used to create a nomogram for individualized prediction. Calibration plots were created to assess predicted vs observed probabilities of seizure recurrence at two and five years after withdrawal. Considering the small number of subjects who had attempted to withdraw ASM (on average 15.3 subjects per cohort; IQR 1-19) we considered an internal-external cross-validation per cohort to be underpowered. To circumvent this, we assessed the external predictive performance of our multivariable model by performing internal-external cross-validations by splitting the 18 cohorts with data on post-withdrawal seizure recurrence into three datasets, balanced on sample size. The model was trained on data from 12/18 cohorts after which predictions were calculated on the 6 holdout cohorts. Based on this, a Concordance statistic (C-statistic) for predictive performance was calculated for each split using the method by Uno et al.[61] The average C-statistic of all splits was calculated, averaged over twenty imputations. The same analyses were performed for recurrence of GTCS after ASM withdrawal, with analyses restricted to subjects who had ever experienced GTCS before withdrawal (n=281).

All statistical analyses were performed in RStudio Version 1.3.1093, using the packages: MICE, metafor, glmer, rms, coxme, rsample, purrr, survminer, tidyverse, ggplot, and survAUC.

**Supplementary references**


1 Asadi-Pooya AA, Hashemzehi Z, Emami M. Predictors of seizure control in patients with juvenile myoclonic epilepsy (JME). *Seizure* 2014; **23**: 889–91.

2 Baykan B, Altindag EA, Bebek N, *et al.* Myoclonic seizures subside in the fourth decade in juvenile myoclonic epilepsy. *Neurology* 2008; **70**: 2123–9.

3 Cação G, Parra J, Mannan S, Sisodiya SM, Sander JW. Juvenile myoclonic epilepsy refractory to treatment in a tertiary referral center. *Epilepsy Behav* 2018; **82**: 81–6.

4 Cerulli Irelli E, Morano A, Barone FA, *et al.* Persistent treatment resistance in genetic generalized epilepsy: A long-term outcome study in a tertiary epilepsy center. *Epilepsia* 2020; **61**: 2452–60.

5 Chowdhury A, Brodie MJ. Pharmacological outcomes in juvenile myoclonic epilepsy: Support for sodium valproate. *Epilepsy Res* 2016; **119**: 62–6.

6 Silvennoinen K, de Lange N, Zagaglia S, *et al.* Comparative effectiveness of antiepileptic drugs in juvenile myoclonic epilepsy. *Epilepsia Open* 2019; **4**: 420–30.

7 Gesche J, Christensen J, Hjalgrim H, Rubboli G, Beier CP. Epidemiology and outcome of idiopathic generalized epilepsy in adults. *Eur J Neurol* 2020; **27**: 676–84.

8 Hernández-Vanegas LE, Jara-Prado A, Ochoa A, *et al.* High-dose versus low-dose valproate for the treatment of juvenile myoclonic epilepsy: Going from low to high. *Epilepsy Behav* 2016; **61**: 34–40.

9 Höfler J, Unterberger I, Dobesberger J, Kuchukhidze G, Walser G, Trinka E. Seizure outcome in 175 patients with juvenile myoclonic epilepsy--a long-term observational study. *Epilepsy Res* 2014; **108**: 1817–24.

10 Japaridze G, Kasradze S, Lomidze G, *et al.* Focal EEG features and therapeutic response in patients with juvenile absence and myoclonic epilepsy. *Clin Neurophysiol* 2016; **127**: 1182–7.

11 Jayalakshmi S, Vooturi S, Bana AK, Sailaja S, Somayajula S, Mohandas S. Factors associated with lack of response to valproic acid monotherapy in juvenile myoclonic epilepsy. *Seizure* 2014; **23**: 527–32.

12 Karakis I, Pathmanathan JS, Chang R, Cook EF, Cash SS, Cole AJ. Prognostic value of EEG asymmetries for development of drug-resistance in drug-naïve patients with genetic generalized epilepsies. *Clin Neurophysiol* 2014; **125**: 263–9.

13 Sun Y, Seneviratne U, Perucca P, *et al.* Generalized polyspike train: An EEG biomarker of drug-resistant idiopathic generalized epilepsy. *Neurology* 2018; **91**: e1822–30.

14 Gürer R, Aydın Ş, Özkara Ç. Outcomes of low-dose valproic acid treatment in patients with juvenile myoclonic epilepsy. *Seizure* 2019; **70**: 43–8.

15 Pietrafusa N, La Neve A, de Palma L, *et al.* Juvenile myoclonic epilepsy: Long-term prognosis and risk factors. *Brain Dev* 2021; **43**: 688–97.

16 Schneider-von Podewils F, Gasse C, Geithner J, *et al.* Clinical predictors of the long-term social outcome and quality of life in juvenile myoclonic epilepsy: 20-65 years of follow-up. *Epilepsia* 2014; **55**: 322–30.

17 Seneviratne U, Boston RC, Cook M, D'Souza W. EEG correlates of seizure freedom in genetic generalized epilepsies. *Neurol Clin Pract* 2017; **7**: 35–44.

18 Syvertsen M, Fløgstad I, Enger U, Landmark CJ, Koht J. Antiepileptic drug withdrawal in juvenile myoclonic epilepsy. *Acta Neurol Scand* 2019; **139**: 192–8.

19 Szaflarski JP, Rackley AY, Lindsell CJ, Szaflarski M, Yates SL. Seizure control in patients with epilepsy: the physician vs. medication factors. *BMC Health Serv Res* 2008; **8**: 264.

20 Vijai J, Cherian PJ, Stlaja PN, Anand A, Radhakrishnan K. Clinical characteristics of a South Indian cohort of juvenile myoclonic epilepsy probands. *Seizure* 2003; **12**: 490–6.

21 Viloria Alebesque A, Bellosta Diago E, Santos Lasaosa S, Mauri Llerda JA. [Juvenile myoclonic epilepsy: long-term prognosis and antiepileptic drug withdrawal]. *An Sist Sanit Navar* 2020; **43**: 43–9.

22 Viswanathan LG, Mundlamuri RC, Raghavendra K, *et al.* Long-Term Seizures Outcome in Juvenile Myoclonic Epilepsy (JME): A Retrospective Cohort Study in an Indian Population. *International Journal of Epilepsy* 2021; **7**: 15–21.

23 Vorderwülbecke BJ, Kowski AB, Kirschbaum A, *et al.* Long-term outcome in adolescent-onset generalized genetic epilepsies. *Epilepsia* 2017; **58**: 1244–50.

24 Zhang Y, Chen J, Ren J, Liu W, Yang T, Zhou D. Clinical features and treatment outcomes of Juvenile myoclonic epilepsy patients. *Epilepsia Open* 2019; **4**: 302–8.

25 Camfield CS, Camfield PR. Juvenile myoclonic epilepsy 25 years after seizure onset: a population-based study. *Neurology* 2009; **73**: 1041–5.

26 Gomez-Ibañez A, McLachlan RS, Mirsattari SM, Diosy DC, Burneo JG. Prognostic factors in patients with refractory idiopathic generalized epilepsy. *Epilepsy Res* 2017; **130**: 69–73.

27 Guaranha MSB, Filho GM de A, Lin K, Guilhoto LMFF, Caboclo LOSF, Yacubian EMT. Prognosis of juvenile myoclonic epilepsy is related to endophenotypes. *Seizure* 2011; **20**: 42–8.

28 Martínez-Juárez IE, Alonso ME, Medina MT, *et al.* Juvenile myoclonic epilepsy subsyndromes: family studies and long-term follow-up. *Brain* 2006; **129**: 1269–80.

29 Mehndiratta MM, Aggarwal P. Clinical expression and EEG features of patients with juvenile myoclonic epilepsy (JME) from North India. *Seizure* 2002; **11**: 431–6.

30 Nicolson A, Appleton RE, Chadwick DW, Smith DF. The relationship between treatment with valproate, lamotrigine, and topiramate and the prognosis of the idiopathic generalised epilepsies. *J Neurol Neurosurg Psychiatry* 2004; **75**: 75–9.

31 Vollmar C, O'Muircheartaigh J, Barker GJ, *et al.* Motor system hyperconnectivity in juvenile myoclonic epilepsy: a cognitive functional magnetic resonance imaging study. *Brain* 2011; **134**: 1710–9.

32 Ashmawi A, Hosny H, Gadallah M, Beghi E. Sleep convulsive seizures predict lack of remission in genetic generalized epilepsies: A retrospective study from a single epilepsy center in Egypt. *Acta Neurol Scand* 2017; **136**: 528–35.

33 Sapio MR, Vessaz M, Thomas P, Genton P, Fricker LD, Salzmann A. Novel carboxypeptidase A6 (CPA6) mutations identified in patients with juvenile myoclonic and generalized epilepsy. *PLoS One* 2015; **10**: e0123180.

34 Healy L, Moran M, Singhal S, O'Donoghue MF, Alzoubidi R, Whitehouse WP. Relapse after treatment withdrawal of antiepileptic drugs for Juvenile Absence Epilepsy and Juvenile Myoclonic Epilepsy. *Seizure* 2018; **59**: 116–22.

35 Sánchez-Zapata P, Zapata-Berruecos JF. [Clinical and psychosocial factors associated with seizure control in patients with juvenile myoclonic epilepsy]. *Rev Neurol* 2019; **69**: 453–60.

36 Gelisse P, Genton P, Thomas P, Rey M, Samuelian JC, Dravet C. Clinical factors of drug resistance in juvenile myoclonic epilepsy. *J Neurol Neurosurg Psychiatry* 2001; **70**: 240–3.

37 Landvogt C, Buchholz H-G, Bernedo V, Schreckenberger M, Werhahn KJ. Alteration of dopamine D2/D3 receptor binding in patients with juvenile myoclonic epilepsy. *Epilepsia* 2010; **51**: 1699–706.

38 Asconapé J, Penry JK. Some clinical and EEG aspects of benign juvenile myoclonic epilepsy. *Epilepsia* 1984; **25**: 108–14.

39 Panayiotopoulos CP, Obeid T, Tahan AR. Juvenile myoclonic epilepsy: a 5-year prospective study. *Epilepsia* 1994; **35**: 285–96.

40 Chakravarty A, Mukherjee A, Roy D. Observations on juvenile myoclonic epilepsy amongst ethnic Bengalees in West Bengal--an Eastern Indian State. *Seizure* 2007; **16**: 134–41.

41 Fernando-Dongas MC, Radtke RA, VanLandingham KE, Husain AM. Characteristics of valproic acid resistant juvenile myoclonic epilepsy. *Seizure* 2000; **9**: 385–8.

42 Wu S-Z, Ye H, Yang X-G, Lu Z-L, Qu Q, Qu J. Case-control pharmacogenetic study of HCN1/HCN2 variants and genetic generalized epilepsies. *Clin Exp Pharmacol Physiol* 2018; **45**: 226–33.

43 Choi H, Detyniecki K, Bazil C, *et al.* Development and validation of a predictive model of drug-resistant genetic generalized epilepsy. *Neurology* 2020; **95**: e2150–60.

44 Chen Y, Chen J, Chen X, *et al.* Predictors of Outcome in Juvenile Myoclonic Epilepsy. *Risk Manag Healthc Policy* 2020; **13**: 609–13.

45 Takagi S, Yamamoto T, Hara K, *et al.* Age at onset and response to antiepileptic drugs in patients with various subtypes of idiopathic generalized epilepsy - preliminary investigation for the prevention of epilepsy. *Epilepsy & Seizure* 2018; **10**: 95–106.

46 Pedersen SB, Petersen KA. Juvenile myoclonic epilepsy: clinical and EEG features. *Acta Neurol Scand* 1998; **97**: 160–3.

47 Canevini MP, Mai R, Di Marco C, *et al.* Juvenile myoclonic epilepsy of Janz: clinical observations in 60 patients. *Seizure* 1992; **1**: 291–8.

48 Clement MJ, Wallace SJ. Juvenile myoclonic epilepsy. *Arch Dis Child* 1988; **63**: 1049–53.

49 Penry JK, Dean JC, Riela AR. Juvenile myoclonic epilepsy: long-term response to therapy. *Epilepsia* 1989; **30 Suppl 4**: S19-23; discussion S24-7.

50 Sharpe C, Buchanan N. Juvenile myoclonic epilepsy: diagnosis, management and outcome. *Med J Aust* 1995; **162**: 133–4.

51 Kleveland G, Engelsen BA. Juvenile myoclonic epilepsy: clinical characteristics, treatment and prognosis in a Norwegian population of patients. *Seizure* 1998; **7**: 31–8.

52 Calleja S, Salas-Puig J, Ribacoba R, Lahoz CH. Evolution of juvenile myoclonic epilepsy treated from the outset with sodium valproate. *Seizure* 2001; **10**: 424–7.

53 Siren A, Eriksson K, Jalava H, Kilpinen-Loisa P, Koivikko M. Idiopathic generalised epilepsies with 3 Hz and faster spike wave discharges: a population-based study with evaluation and long-term follow-up in 71 patients. *Epileptic Disord* 2002; **4**: 209–16.

54 Van Buuren S, Groothuis-Oudshoorn K. mice: Multivariate imputation by chained equations in R. *J Stat Softw* 2011; **45**: 1–67.

55 Cummings P. Missing data and multiple imputation. *JAMA Pediatr* 2013; **167**: 656–61.

56 Azur MJ, Stuart EA, Frangakis C, Leaf PJ. Multiple imputation by chained equations: what is it and how does it work? *Int J Methods Psychiatr Res* 2011; **20**: 40–9.

57 Vergouw D, Heymans MW, van der Windt DAWM, *et al.* Missing data and imputation: a practical illustration in a prognostic study on low back pain. *J Manipulative Physiol Ther* 2012; **35**: 464–71.

58 Moons KGM, Donders RART, Stijnen T, Harrell FE Jr. Using the outcome for imputation of missing predictor values was preferred. *J Clin Epidemiol* 2006; **59**: 1092–101.

59 van Ginkel JR, Linting M, Rippe RCA, van der Voort A. Rebutting Existing Misconceptions About Multiple Imputation as a Method for Handling Missing Data. *J Pers Assess* 2020; **102**: 297–308.

60 Patricia M, Grambsch G, Terry M, Therneau T. 'Proportional hazards tests and diagnostics based on weighted residuals.' *Biometrika* 1995; **82**: 668–668.

61 Uno H, Cai T, Pencina MJ, D'Agostino RB, Wei LJ. On the C-statistics for evaluating overall adequacy of risk prediction procedures with censored survival data. *Stat Med* 2011; **30**: 1105–17.
